# Identification of candidate genes related to salt tolerance of the secretohalophyte *Atriplex canescens* by transcriptomic analysis

**DOI:** 10.1186/s12870-019-1827-6

**Published:** 2019-05-22

**Authors:** Huan Guo, Le Zhang, Yan-Nong Cui, Suo-Min Wang, Ai-Ke Bao

**Affiliations:** State Key Laboratory of Grassland Agro-ecosystems, Key Laboratory of Grassland Livestock Industry Innovation, Ministry of Agriculture and Rural Affairs, College of Pastoral Agriculture Science and Technology, Lanzhou University, Lanzhou, 730020 People’s Republic of China

**Keywords:** Halophyte, *Atriplex canescens*, Salt tolerance, Transcriptomic analysis, Differentially expressed gene

## Abstract

**Background:**

*Atriplex canescens* is a typical C_4_ secretohalophyte with salt bladders on the leaves. Accumulating excessive Na^+^ in tissues and salt bladders, maintaining intracellular K^+^ homeostasis and increasing leaf organic solutes are crucial for *A. canescens* survival in harsh saline environments, and enhanced photosynthetic activity and water balance promote its adaptation to salt. However, the molecular basis for these physiological mechanisms is poorly understood. Four-week-old *A. canescens* seedlings were treated with 100 mM NaCl for 6 and 24 h, and differentially expressed genes in leaves and roots were identified, respectively, with Illumina sequencing.

**Results:**

In *A. canescens* treated with 100 mM NaCl, the transcripts of genes encoding transporters/channels for important nutrient elements, which affect growth under salinity, significantly increased, and genes involved in exclusion, uptake and vacuolar compartmentalization of Na^+^ in leaves might play vital roles in Na^+^ accumulation in salt bladders. Moreover, NaCl treatment upregulated the transcripts of key genes related to leaf organic osmolytes synthesis, which are conducive to osmotic adjustment. Correspondingly, aquaporin-encoding genes in leaves showed increased transcripts under NaCl treatment, which might facilitate water balance maintenance of *A. canescens* seedlings in a low water potential condition. Additionally, the transcripts of many genes involved in photosynthetic electron transport and the C_4_ pathway was rapidly induced, while other genes related to chlorophyll biosynthesis, electron transport and C_3_ carbon fixation were later upregulated by 100 mM NaCl.

**Conclusions:**

We identified many important candidate genes involved in the primary physiological mechanisms of *A. canescens* salt tolerance. This study provides excellent gene resources for genetic improvement of salt tolerance of important crops and forages.

**Electronic supplementary material:**

The online version of this article (10.1186/s12870-019-1827-6) contains supplementary material, which is available to authorized users.

## Background

Salinity is one of the most severe abiotic factors threatening agricultural productivity and ecological environment throughout the world [2, 12]. Approximately half of irrigated lands in the world are threatened by salinity [43]. The expansion of soil salinization and the increasing human population are forcing agricultural production into marginal areas [44]. Soil salinization can significantly reduce the yield and quality of crops by resulting in a series of metabolic disturbances due to ionic toxicity, physiological drought and nutrient deficiency [14, 63]. Therefore, improving the salt tolerance of plants, especially traditional crops and forages, is currently an urgent issue, since most of these species are glycophytes with weak salt tolerance [43, 51]. In contrast, halophytic species have evolved multiple adaptation strategies to deal with harsh saline environments [12, 44]. Learning from halophytes, understanding the mechanisms underlying plant response to salinity and thereby identifying key genes related to salt tolerance will contribute to breeding crops with salt tolerance [61].

Halophytes can be divided into three types based on their adaptive strategies to saline environments: (i) pseudohalophytes maintain a low Na^+^ level by limiting Na^+^ uptake [52], (ii) euhalophytes compartmentalize Na^+^ into swollen internal vacuoles to alleviate Na^+^ toxicity in the cytosol [14, 56] and (iii) secretohalophytes exclude excessive Na^+^ from secreting structures (salt glands or salt bladders) on the surface of stems and/or leaves [11, 44]. For example, *Reaumuria trigyna* and *Limonium bicolor* are capable of secreting Na^+^ via their multicellular salt glands, but excreting little K^+^ to maintain high K^+^/Na^+^ ratio in the shoots [8, 62]. *Mesembryanthemum crystallinum*, *Chenopodium quinoa* and *Atriplex* species deposit a large amount of Na^+^ in epidermal bladder cells (EBCs) to improve salt tolerance of plants [26, 39]. Approximately half of all halophyte plants possess salt bladders, which segregate excessive Na^+^ away from metabolically active organs in the growing plant body [13, 44]; hence, these plants are likely to be potential species for saline soil amelioration and improvement of salt tolerance in important crops.

*Atriplex canescens* (four-wing saltbush), a C_4_ perennial semi-evergreen woody shrub with excellent adaptability to salinity and drought, is a typical secretohalophyte with salt bladders that is widely distributed in saline and arid regions [22]. This species is commonly planted in highway medians and on road shoulders, slopes, and other disturbed areas for erosion control and reclamation of marginal lands, and it can be used as a landscape plant in the arid regions of northern China; moreover, *A. canescens* is an attractive fodder crop for most livestock because of its high palatability and nutritional value [17, 40]. Early research findings showed that *A. canescens* could grow along a salinity gradient from 72 to 2017 mol/m^3^ NaCl in the root zone and accumulated more Na^+^ than K^+^ for osmotic adjustment (OA) at relatively low salinities [17, 18]. Our previous study revealed that moderate salinity (100 mM NaCl) could stimulate the growth of *A. canescens* and high salinity (400 mM NaCl) had no significant effect on its growth [40]. Under saline conditions, *A. canescens* can enhance photosynthetic capacity, accumulate more Na^+^ in tissues and salt bladders, maintain leaf K^+^ homeostasis, and use inorganic ions as well as organic osmolytes for OA, which may contribute to water balance in the plant [40]. Our latest investigation showed that the addition of 100 mM NaCl effectively alleviated the adverse impact of drought on the growth of *A. canescens* by increasing the accumulation of solutes (Na^+^, free proline, betaine and soluble sugar) in leaves as well as the net photosynthetic rate and water content (Guo H. and Bao A.K., unpublished data). All of these results indicate that the transport of Na^+^ and K^+^, the accumulation of organic solutes, the improvement of photosynthetic activity and leaf hydration are vital strategies for *A. canescens* adaptation to saline environments. Nonetheless, the possible molecular basis of these important physiological mechanisms is poorly understood owing to the absence of genomic data in *A. canescens*.

High-throughput RNA sequencing has been widely used to investigate the molecular processes related to adaptive responses to abiotic stresses and to identify stress-resistance candidate genes by analyzing differences in transcript abundance [57]. In this work, transcriptomes of *A. canescens* were generated by Illumina assembly technology to lay the foundation for exploring the potential salt tolerance mechanisms of this species. In addition, the genes showing significant transcriptional changes in *A. canescens* under NaCl treatment were then identified by comparing the gene transcript profiles in leaves and roots between salt-treated and control plants by using a tag-based digital gene expression (DGE) system, mainly focusing on identifying the candidate genes related to ion transport, organic osmolyte accumulation, water transport and photosynthesis.

## Results

### Transcriptome sequencing, de novo assembly and unigene functional annotation

A total of 13.37 and 13.41 Gb clean bases were generated from the leaves and roots of *A. canescens* by Illumina HiSeq sequencing, respectively (Additional file 1: Table S1). Then, 207.20 Mb raw reads were yielded from leaves and 210.00 Mb raw reads were yielded from roots through high-throughput sequencing (Additional file 1: Table S1). After filtering, a total of 133.70 and 134.12 Mb clean reads were generated from the leaves and roots, respectively, coupled with a Q20 score greater than 97 and 0.00% Ns (Additional file 1: Table S1). All of these results indicated that the output and quality of transcriptome sequencing were adequate for subsequent analysis.

Paired-end information was used to join contigs into scaffolds and further assembly^23^, and 54,611 and 59,582 unigenes, with a mean length of 912 and 696 bp, respectively, were generated from the leaves and roots (Table 1). Then, 70,571 all-unigene sequences were acquired, with a mean length of 961 bp, N50 of 1647 bp and GC percentage of 40.01%, after further assembly of the unigenes from leaves and roots (Table 1). The size distribution is shown in Additional file 1: Figure S1, and the lengths of 24,205 unigenes were more than 1000 bp.

**Table 1:** Overview of de novo sequence assembly

Unigenes	Total number	Total length (bp)	Mean length (bp)	N50 (bp)	GC (%)
Leaves	54,611	49,833,011	912	1547	40.21
Roots	59,582	76,399,446	696	1332	40.19
All	70,571	67,859,996	961	1647	40.01

Then, 44,121 unigenes (62.52% of the 70,571 unigenes) were annotated to known genes in 7 databases, namely, the Nr, Nt, Swiss-Prot, KEGG, COG, InterPro and GO databases (Additional file 1: Table S2). Functional annotation was not obtained for 37.48% of the unigenes due to the absence of genomic data in *A. canescens* and close-related species. Among these annotated unigenes, 26,021 unigenes annotated in the COG database in terms of sequence homology were classified into 25 functional clusters and 37,395 unigenes annotated with GO terms were grouped into 3 main GO categories with 52 subcategories (Additional file 1:Figures S2 and S3).

### Differentially expressed genes (DEGs) in *A. canescens* under NaCl treatment

Eight independent cDNA libraries (CL6, CR6, SL6, SR6, CL24, CR24, SL24 and SR24) were sequenced, and approximately 22 million raw reads were generated in each library; after filtering low-quality reads, we obtained 21 million clean reads in each library (data not shown), more than 72% of which could be mapped to the transcriptome reference database (data not shown).

**Fig. 1:**
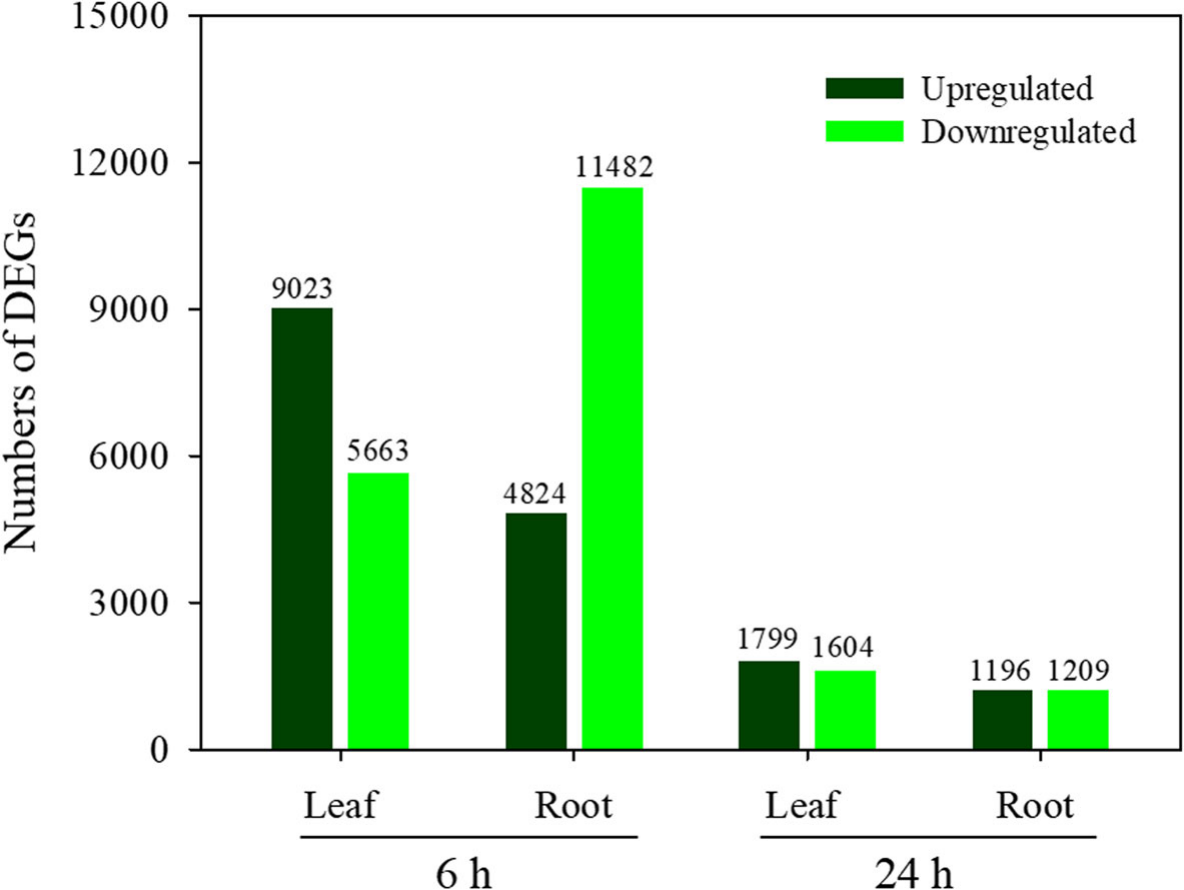
Numbers of differentially expressed genes (DEGs) in leaves and roots of *A. canescens* under 100 mM NaCl for 6 and 24 h. A FDR < 0.001 and an absolute value of the log_2_Ratio > 1 were used as the thresholds to determine significant differences in gene expression

The DEGs in *A. canescens* were analyzed by comparing the 100 mM NaCl treatment with the control. When plants were subjected to the 100 mM NaCl treatment for 6 h, 14,686 and 16,306 DEGs were found in the leaves and roots, respectively, using the thresholds of FDR < 0.001 and |log_2_Ratio| > 1 (Fig. 1). Among these DEGs, 9023 and 4824 DEGs were upregulated, including 1768 and 1031 DEGs that were expressed in the leaves and roots of plants in the 100 mM NaCl treatment but almost not expressed in the control (the FPKM value was 0.01 in control plants, the same below), respectively (Fig. 1). Interestingly, the number of upregulated DEGs was much greater than that of downregulated DEGs in the leaves; conversely, the number of upregulated DEGs was much lower than that of downregulated DEGs in the roots. In addition, 3403 and 2405 DEGs (including 1799 and 1196 upregulated DEGs) were identified in the leaves and roots of plants treated to 100 mM NaCl for 24 h, respectively, which was far fewer than the number identified in plants exposed to the treatment for 6 h. Among these upregulated DEGs, 629 and 426 DEGs were expressed in the leaves and roots of plants in the 100 mM NaCl treatment while almost not expressed in the control, respectively (Fig. 1).

### DEGs related to ion, glucose and oxygen transport

Firstly, the DEGs related to ion transport especially Na^+^ transport into salt bladders were identified under 100 mM NaCl treatment since accumulating a great quantity of Na^+^ in salt bladders and maintaining intracellular ion homeostasis are vital strategies for *A. canescens* adaptation to saline conditions.

**Fig. 2:**
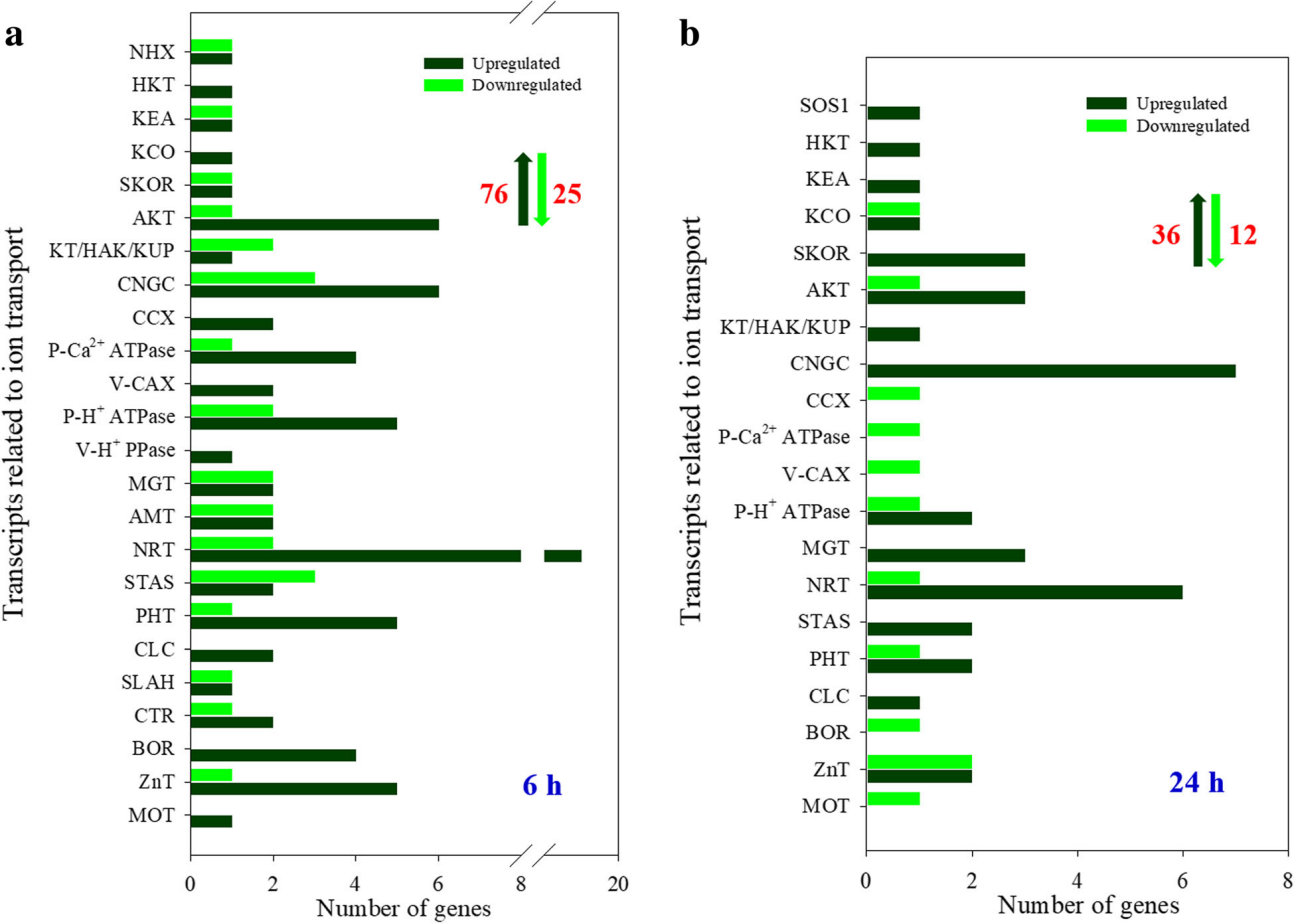
The DEGs related to ion transport in leaves of *A. canescens* under 100 mM NaCl for 6 (**a**) and 24 h (**b**). NHX: tonoplast Na^+^/H^+^ antiporter, SOS1: plasma membrane Na^+^/H^+^ antiporter, HKT: high-affinity K^+^ transporter, KEA: K^+^ efflux antiporter, KCO: calcium-activated outwardly rectifying potassium channel, SKOR: stelar K^+^ outwardly rectifying channel, AKT: inwardly rectifying K^+^ channel, KT/HAK/KUP: K^+^ transporter, CNGC: cyclic nucleotide-gated channel, CCX: cation/Ca^2+^ exchanger, P-Ca^2+^ ATPase: plasma membrane Ca^2+^ ATPase, V-CAX: vacuolar cation/H^+^ exchanger, P-H^+^ ATPase: plasma membrane H^+^ ATPase, V-H^+^ PPase: vacuolar H^+^ PPase, MGT: Mg^2+^ transporter, AMT: NH_4_^+^ transporter, NRT: NO_3_^−^ transporter, STAS: SO_4_^2−^ transporter, PHT: PO_4_^3−^ transporter, CLC: vacuolar Cl^−^/H^+^ exchanger, SLAH: Slow type anion channel, CTR: Cu^2+^ transporter, BOR: BO^3−^ transporter, ZnT: Zn^3+^ transporter, MOT: MoO_4_^2−^ transporter. The up and down arrows indicate the total number of up and downregulated DEGs, respectively. The same below

In leaves, the number of upregulated DEGs associated with ion transport was much larger than that of downregulated DEGs when plants were treated with 100 mM NaCl for either 6 h or 24 h (Fig. 2). After plants were treated with 100 mM NaCl for 6 h, 76 DEGs were upregulated, which included important transcripts related to Na^+^ (including *NHX* and *HKT*), K^+^ (such as *AKT* and *SKOR*), Ca^2+^ (*CNGC*, *CCX* and *P-Ca*^*2+*^ *ATPase*), Mg^2+^ (*MGT*), and NH_4_^+^ (*AMT*) transport and the anion transport of NO^3−^ (*NRT*), PO_4_^3−^ (*PHT*), SO_4_^2−^ (*STAS*), Cl^−^ (*CLC* and *SLAH*) and several important micronutrients (such as Zn, Mo, B and Cu) (Fig. 2a; Additional file 1: Table S3). Some genes encoding plasma membrane H^+^-ATPases (P-H^+^ ATPase) and vacuolar H^+^-pyrophosphatases (V-H^+^ PPase) were also upregulated (Fig. 2a). The number of DEGs was significantly lower under 100 mM NaCl for 24 h than under 100 mM NaCl for 6 h (Fig. 2b). Among these upregulated genes, two transcripts associated with Na^+^ transport (*SOS1* and *HKT1*) were upregulated, and the other upregulated DEGs were mainly SKOR, AKT, CNGC, MGT and NRT transport protein family genes, which are related to nutrient element transport (Fig. 2b; Additional file 1: Table S4). In addition, among these DEGs related to K^+^ transport in leaves, 9 DEGs were significantly upregulated under 100 mM NaCl but not under control conditions for either 6 or 24 h (Table 2).

**Table 2:** The upregulated DEGs related to K^+^ transport in roots and leaves of *A. canescens* under 100 mM NaCl but not under control condition for either 6 or 24 h

Roots		Leaves	
Gene ID	Homologous gene	Gene ID	Homologous gene
CL5980.Contig1_All	Potassium channel AKT2/3	Unigene23528_All	Potassium channel AKT1
CL6985.Contig2_All	Inwardly rectifying K^+^ channel AKT1	Unigene25880_All	Potassium channel AKT1
Unigene23969_All	Potassium channel AKT1	CL6985.Contig2_All	Inwardly rectifying K^+^ channel AKT1
Unigene39479_All	Potassium channel SKOR	Unigene39479_All	Potassium channel SKOR
Unigene12729_All	Cyclic nucleotide-gated ion channel CNGC4	CL7131.Contig1_All	Cyclic nucleotide-gated ion channel CNGC18
Unigene19313_All	Cyclic nucleotide-gated ion channel CNGC2	CL1899.Contig4_All	Cyclic nucleotide-gated ion channel CNGC14
CL2081.Contig2_All	Potassium transporter HAK13	CL2081.Contig2_All	Potassium transporter HAK13
Unigene7902_All	Two-pore potassium channel KCO1	Unigene23223_All	K^+^ efflux antiporter KEA2
		CL6946.Contig2_All	Two-pore potassium channel KCO1

**Fig. 3:**
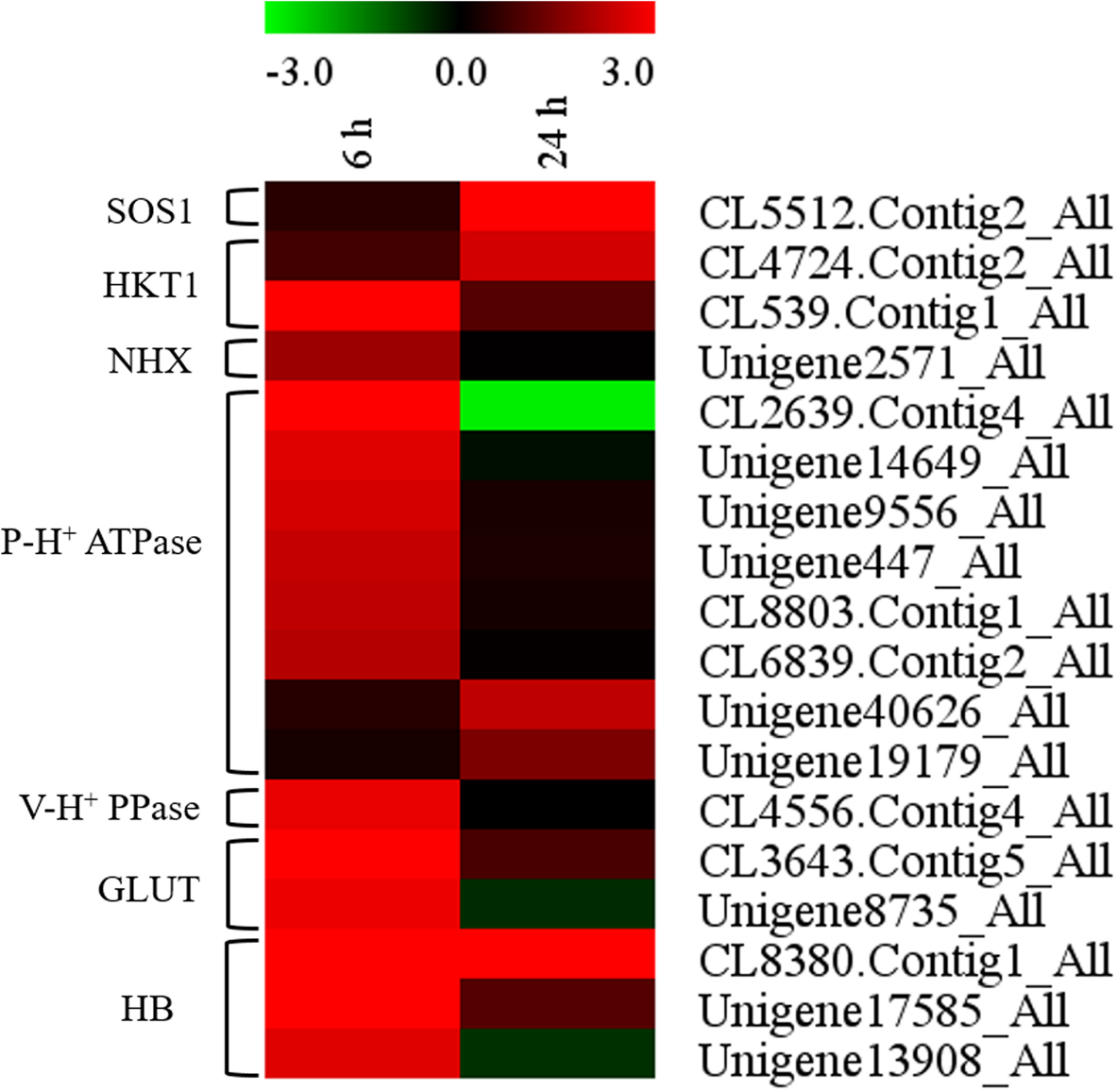
Heatmap of DEGs related to Na^+^, H^+^, glucose transporters and hemoglobin in leaves of *A. canescens* under 100 mM NaCl for 6 and 24 h

The epidermal bladder cell (EBC) together with stalk cell (SC) and epidermal cell (EC) constitute the EC-SC-EBC complex [44]. The Na^+^ sequestration in the high vacuolization of salt bladder is achieved by four times Na^+^ transport through plasma membrane and one time Na^+^ transport through tonoplast [65]. A latest study on another halophyte *C. quinoa* has proposed that the outward Na^+^ movement across plasma membrane of EC and SC is mediated by SOS1; the inward Na^+^ movement across plasma membrane of SC and EBC is mediated by HKT1, coupled with P-H^+^ ATPase providing proton gradients and membrane potential; the Na^+^ movement into vacuole of EBC is mediated by NHX, coupled with V-H^+^ PPase providing proton gradients and membrane potential [4]. Therefore, we further analyzed the transcripts pattern of *SOS1*, *HKT1*, *NHX*, *P-H*^*+*^ *ATPase* and *V- H*^*+*^ *PPase* in leaves of *A. canescens* exposed to 100 mM NaCl for 6 and 24 h. The results showed that the upregulated DEGs in leaves under salt treatment included 1 *SOS1*, 2 *HKT1s*, 1 *NHX*, 8 *P-H*^*+*^ *ATPases* and 1 *V-H*^*+*^ *PPase* (Fig. 3), indicating these genes may play vital roles in Na^+^ sequestration in salt bladders of *A. canescens*. It was also demonstrated that monosaccharides are transported from mesophyll cells to bladder cells by the glucose transporters (GLUTs) and enter the tricarboxylic acid cycles, and hemoglobin (HB) is conducive to facilitate oxygen diffusion into stalk and bladder cells and the oxygen is used for oxidative phosphorylation, these two processes both help to produce ATP for Na^+^ sequestration [65]. Our results showed that upregulated DEGs in leaves under 100 mM NaCl also included 2 GLUTs and 3 HBs (Fig. 3).

**Fig. 4:**
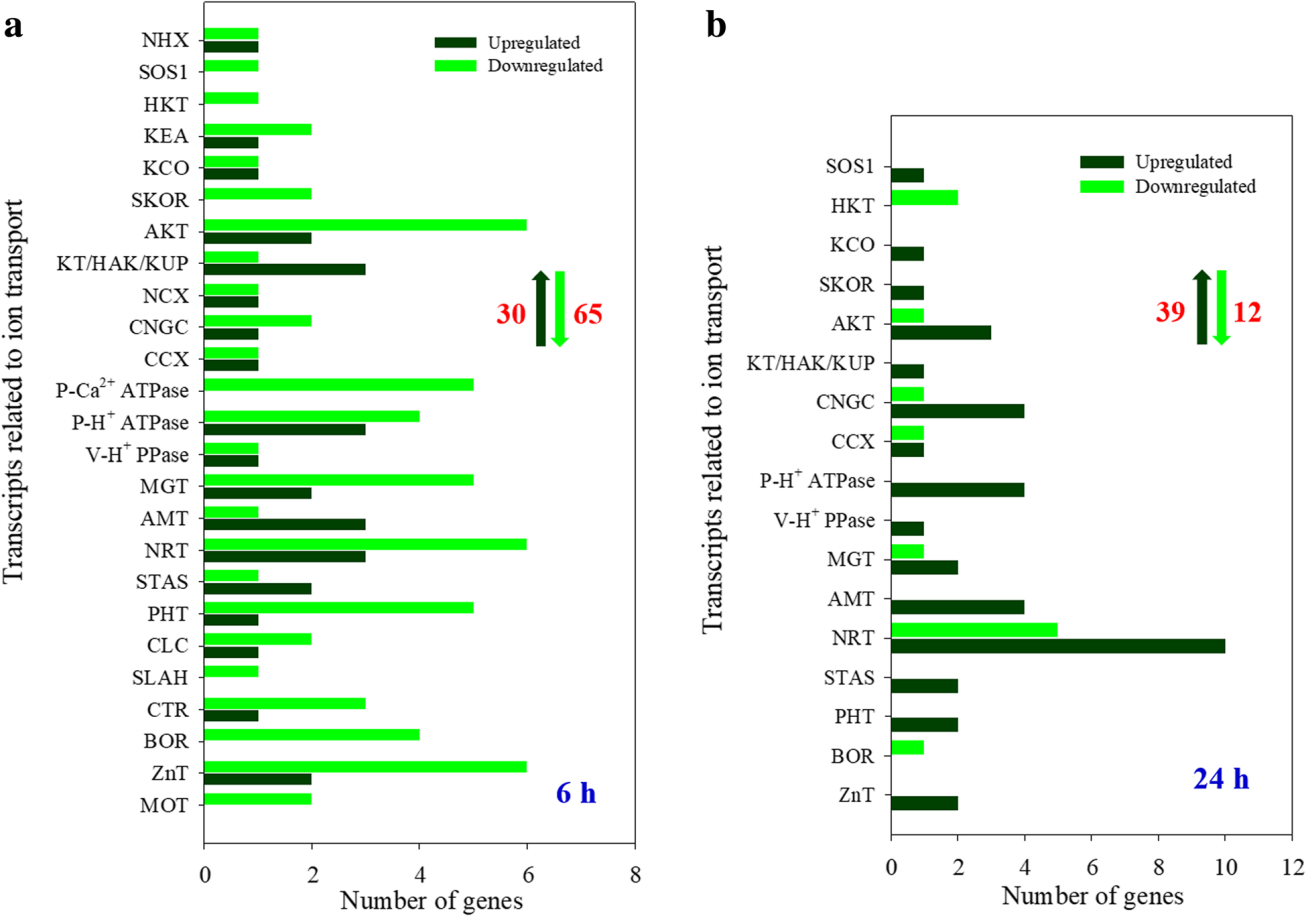
The DEGs related to ion transport in roots of *A. canescens* under 100 mM NaCl for 6 (**a**) and 24 h (**b**)

In roots, the number of upregulated DEGs was much lower than that of downregulated DEGs when plants were treated with 100 mM NaCl for 6 h (Fig. 4a). These upregulated DEGs included important genes involved in the transport of Na^+^, K^+^, Ca^2+^ (such as *NHX*, *KT/HAK/KUP* and *CNGC*), Mg^2+^ (*MGT*), NH_4_^+^ (*AMT*), NO^3−^ (*NRT*), SO_4_^2−^ (*STAS*), PO_4_^3−^ (*PHT*) and several important micronutrients, and 4 H^+^-pump genes were also upregulated (Fig. 4a; Additional file 1: Table S5). After plants were treated with 100 mM NaCl for 24 h, the number of upregulated DEGs remained stable, but the number of downregulated DEGs was significantly lower than that under treatment for 6 h (Fig. 4a and b). Most of these upregulated genes are involved in the transport of K^+^ (*AKT* and *KT/HAK/KUP*), Ca^2+^ (*CNGC*), NH_4_^+^ (*AMT*) and NO^3−^ (*NRT*); additionally, 4 *P-H*^*+*^ *ATPases* and 1 *V-H*^*+*^ *PPase* genes were upregulated (Fig. 4b; Additional file 1: Table S6). More importantly, the transcripts levels of 8 DEGs involved in K^+^ transport were upregulated in roots under 100 mM NaCl but not under control conditions for either 6 or 24 h (Table 2).

### DEGs related to organic osmolyte synthesis

The accumulation of highly compatible solutes in leaves is another important reason why *A. canescens* has a high tolerance to harsh saline conditions. Thus, the DEGs related to the synthesis of organic osmolytes (including proline, betaine and soluble sugar) in the leaves of *A. canescens* under 100 mM NaCl were identified.

**Fig. 5:**
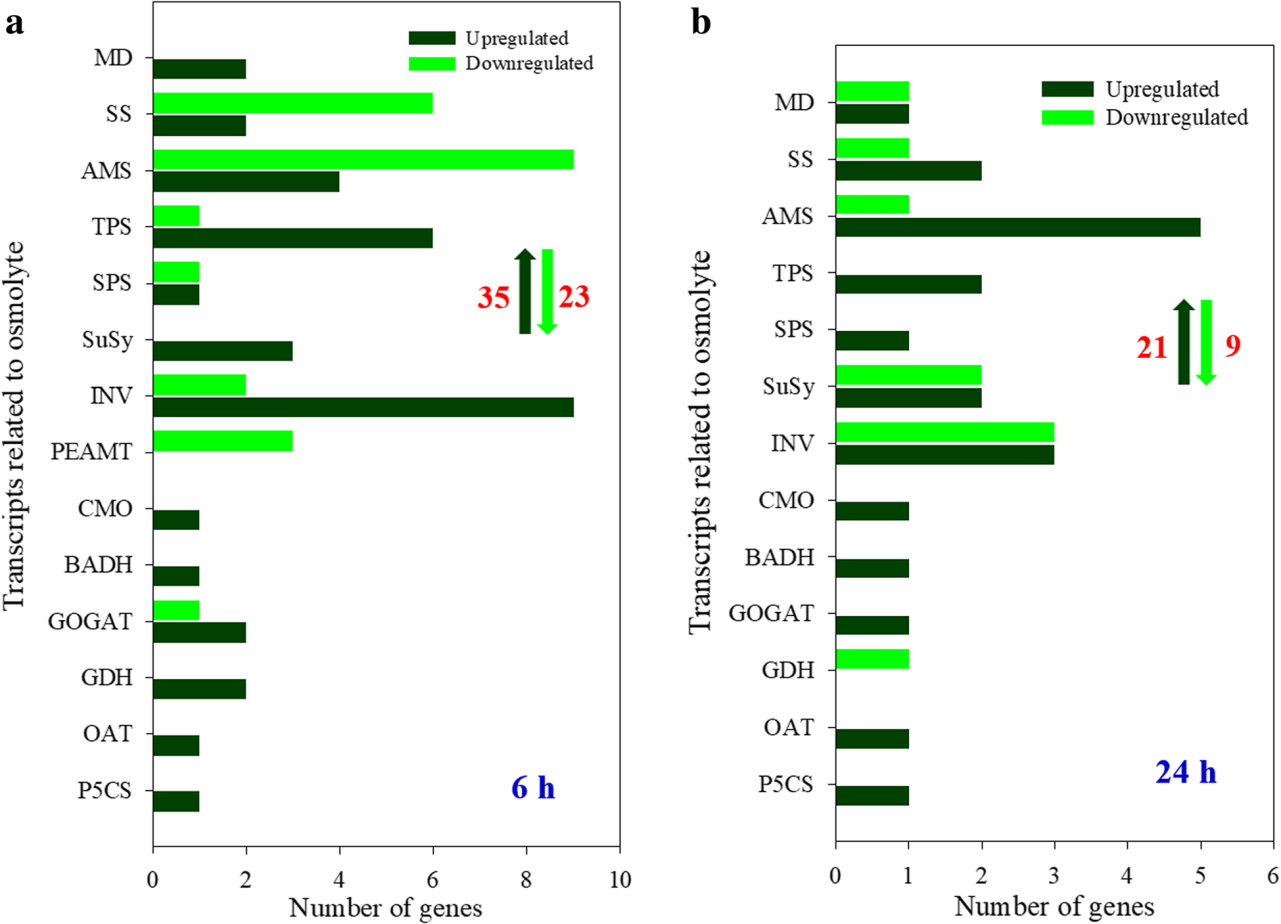
The DEGs related to organic osmolytes synthesis in leaves of *A. canescens* under 100 mM NaCl for 6 (**a**) and 24 h (**b**). P5CS: Δ1-pyrroline-5-carboxylate synthetase, OAT: ornithine aminotransferase, GDH: glutamate dehydrogenase, GOGAT: glutamate synthase, BADH: betaine aldehyde dehydrogenase, CMO: choline monooxygenase, PEAMT: phosphoethanolamine N-methyltransferase, INV: invertase, SuSy: sucrose synthase, SPS: sucrose-phosphate synthase, TPS: trehalose-phosphate synthase, AMS: amylase, SS: starch synthase, MD: mannitol dehydrogenase. The up and down arrows indicate the total number of up and downregulated DEGs, respectively

There were 35 and 21 upregulated DEGs and 23 and 9 downregulated DEGs related to organic osmolytes accumulation in leaves when plants were treated with 100 mM NaCl for 6 and 24 h, respectively (Fig. 5a and b). When plants were treated with 100 mM NaCl for 6 h, the upregulated DEGs included important enzyme genes related to proline production (*P5CS*, *OAT*, *GDH* and *GOGAT*), betaine synthesis (*BADH* and *CMO*) and soluble carbohydrate accumulation (such as *INV*, *SuSy* and *TPS*) (Fig. 5a; Additional file 1: Table S7). Likewise, many genes encoding enzymes related to the synthesis of organic osmolytes mentioned above were also upregulated by treatment with 100 mM NaCl for 24 h (Fig. 5b; Additional file 1: Table S8). Moreover, the transcripts levels of 2, 2 and 9 genes involved in accumulating proline, betaine and soluble sugar, respectively, showed a significant increase in leaves under 100 mM NaCl treatment for both 6 and 24 h (Table 3).

**Table 3:** The upregulated DEGs related to organic osmolytes synthesis in leaves of *A. canescens* under 100 mM NaCl for both 6 and 24 h

Involved process	Gene ID	Homologous gene
Proline production	Unigene27678_All	Pyrroline-5-carboxylate synthetase P5CS
	CL9819.Contig4_All	Glutamate synthase GOGAT
Betaine synthesis	CL8998.Contig2_All	Betaine aldehyde dehydrogenase BADH
	CL1302.Contig2_All	Choline monooxygenase CMO
Sucrose accumulation	CL879.Contig1_All	Exocellular acid invertase INV
	CL796.Contig1_All	Sucrose synthase SuSy2
	CL1644.Contig3_All	Sucrose synthase SuSy7
	CL9220.Contig3_All	Sucrose-phosphate synthase SPS
Amylolysis	CL2655.Contig3_All	β-amylase AMS4
	CL6110.Contig1_All	β-amylase AMS7
Trehalose synthesis	Unigene1043_All	Trehalose-phosphate synthase TPS
Mannitol synthesis	CL4146.Contig3_All	Mannitol dehydrogenase MD
	CL5740.Contig4_All	Mannitol dehydrogenase MD

### DEGs related to water transport

**Fig. 6:**
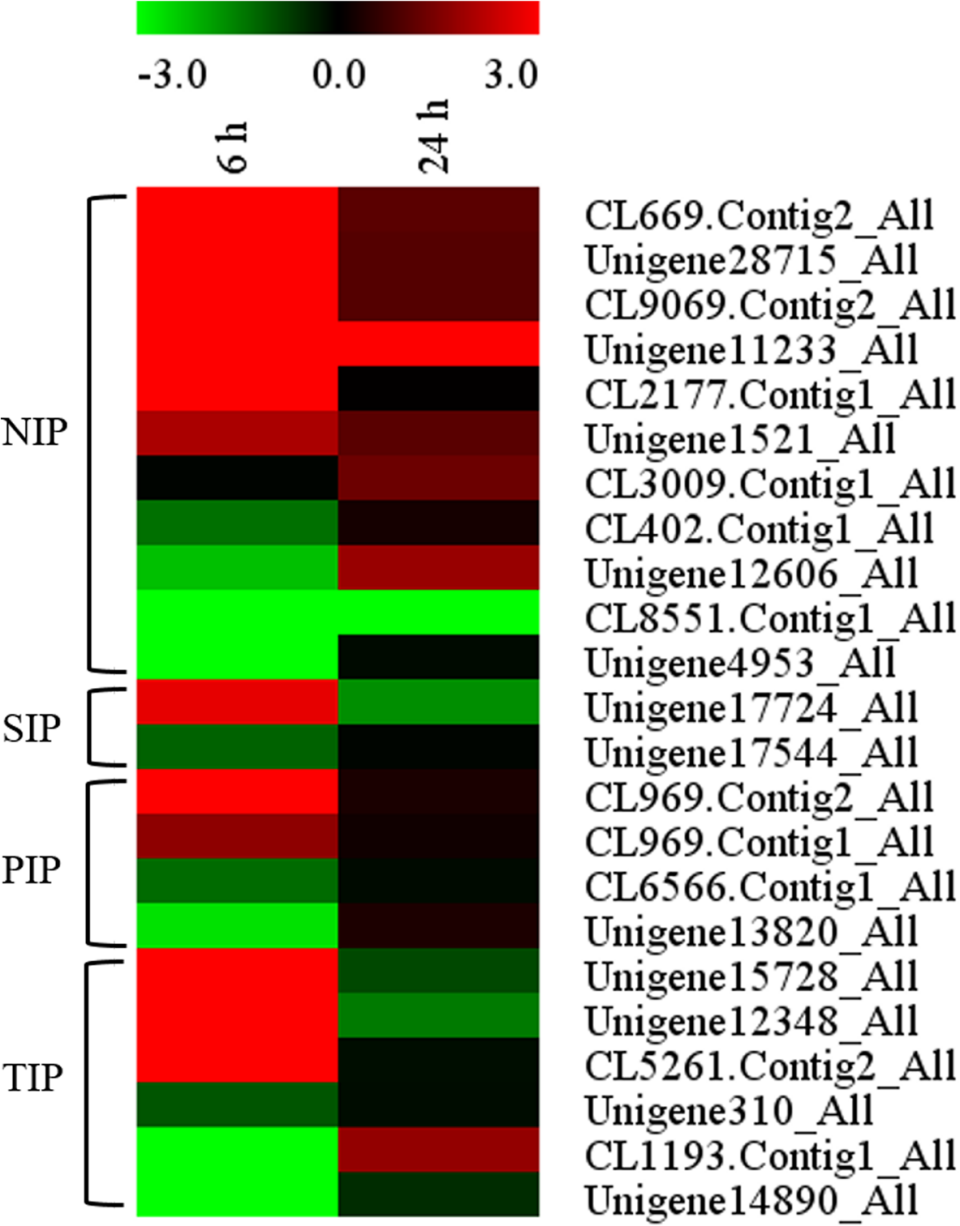
Heatmap of the transcript level of genes related to aquaporin in *A. canescens* under 100 mM NaCl treatment for 6 and 24 h. NIP: nodulin-like intrinsic protein, SIP: small basic intrinsic protein, PIP: plasma membrane intrinsic protein, TIP: tonoplast intrinsic protein. The same below

To understand how *A. canescens* maintains water balance under saline conditions, we analyzed the DEGs related to aquaporin (AQP) in the leaves of *A. canescens* exposed to the 100 mM NaCl treatment. In total, 24 AQP genes were found in *A. canescens* under 100 mM NaCl, including 11 *NIP*, 2 *SIP*, 4 *PIP* and 6 *TIP* genes (Fig. 6). After treatment with 100 mM NaCl for 6 h, there were 6, 1, 2 and 3 upregulated DEGs categorized into the gene families listed above, respectively, and when plants were treated with 100 mM NaCl for 24 h, the number of upregulated DEGs was obviously lower than that in plants treated for 6 h (Fig. 6). Among these AQP genes, interestingly, 12 genes showed upregulated expression in leaves but were downregulated in the roots under 100 mM NaCl for 6 h. For example, the transcripts of the tonoplast aquaporin gene *TIP2;2* (Unigene15728_All) dramatically increased by more than 14 times in leaves but decreased by 12 times in roots (Table 4).

**Table 4:** Aquaporin genes upregulated in leaves but downregulated in roots of *A. canescens* under 100 mM NaCl for 6 h. “Fold change in leaves” equals log_2_ (FPKM-SL6/FPKM-CL6) and “Fold change in roots” equals log_2_ (FPKM-SR6/FPKM-CR6)

Gene ID	Homlogous gene	Fold change in leaves	Fold change in roots
CL669.Contig2_All	NIP4;1	11.62	−11.17
Unigene28715_All	NIP5;1	9.85	−3.84
CL9069.Contig2_All	NIP5;1	7.71	−8.41
Unigene11233_All	NIP5;1	6.70	−5.64
CL2177.Contig1_All	NIP1;2	4.05	−4.17
Unigene1521_All	NIP1;2	2.01	−1.76
Unigene17724_All	SIP1;2	2.72	−1.77
CL969.Contig2_All	PIP2;1	3.04	−3.11
CL969.Contig1_All	PIP1;2	1.69	−1.02
Unigene15728_All	TIP2;2	14.24	−12.17
Unigene12348_All	TIP4;1	4.89	−4.87
CL5261.Contig2_All	TIP1;3	4.87	−4.84

### DEGs related to photosynthesis

**Fig. 7:**
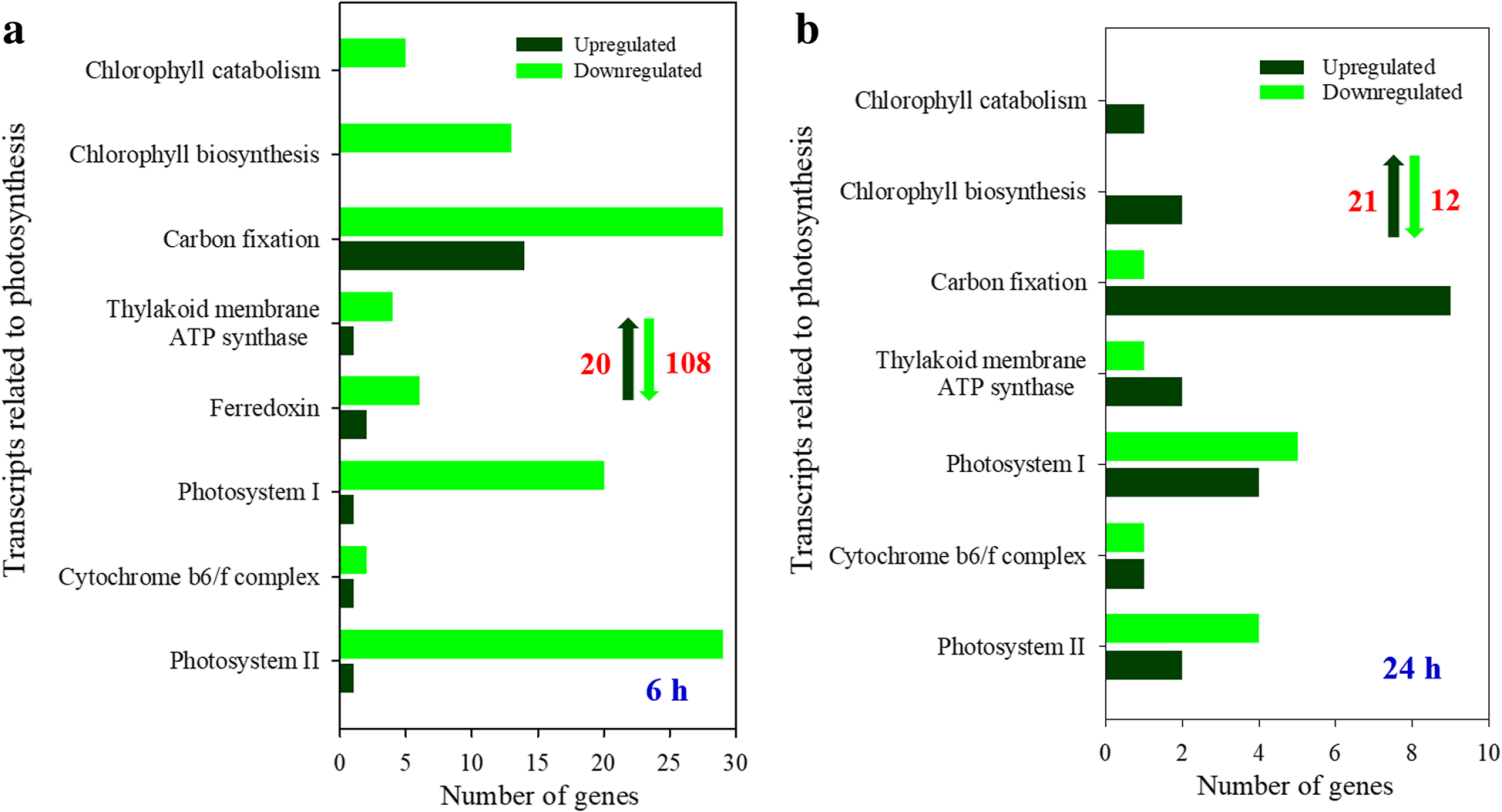
The DEGs related to photosynthesis of *A. canescens* under 100 mM NaCl for 6 (**a**) and 24 h (**b**)

Higher photosynthetic capacity is also an important strategy for *A. canescens* adaptation to saline environments. Therefore, the DEGs related to photosynthesis under 100 mM NaCl were identified. The number of upregulated DEGs was much lower than that of downregulated DEGs after treatment with 100 mM NaCl for 6 h, and the upregulated DEGs included 1, 1, 1, 2, 1 and 14 genes related to photosystem II, the component of cytochrome *b6/f* complex, photosystem I, ferredoxin, thylakoid membrane ATP synthase and the enzymes involved in carbon fixation, respectively (Fig. 7a; Additional file 1: Table S9). When plants were treated with 100 mM NaCl for 24 h, the number of upregulated DEGs remained stable, and the number of downregulated DEGs decreased sharply from 108 to 12 compared with that under treatment for 6 h, and there were 2, 1, 4, 2, 9, 2 and 1 upregulated DEGs related to the component of photosystem II, cytochrome *b6/f* complex, photosystem I, ATP synthase, enzymes related to carbon fixation, chlorophyll biosynthesis and chlorophyll catabolism, respectively (Fig. 7b; Additional file 1: Table S10). Then, we analyzed these upregulated DEGs in depth and found that 5 and 9 upregulated DEGs (including 2 phosphoenolpyruvate carboxylase, 2 malate dehydrogenase, 2 malic enzyme and 3 aspartate aminotransferase) were involved in the process of photosynthetic electron transport and the C_4_ carbon fixation pathway, respectively, under 100 mM NaCl treatment for 6 h (Table 5), indicating that these genes are likely to provide prerequisites for the improvement of photosynthetic capacity of *A. canescens* under saline conditions. In addition, the transcripts of 2, 6 and 4 DEGs involved in the process of chlorophyll biosynthesis, photosynthetic electron transport and the enzymes associated with carbon fixation, respectively, were significantly upregulated after treated with 100 mM NaCl for 24 h but not for 6 h (Table 5).

**Table 5:** The upregulated DEGs related to photosynthesis of *A. canescens* under 100 mM NaCl. The genes upregulated at 6 h are shaded in red, and the genes upregulated at 24 h but not at 6 h are shaded in blue

Involved process	Gene ID	Homologous gene	log_2_ Ratio (SL6/CL6)	log_2_ Ratio (SL24/CL24)
Electron transport	Unigene31059_All	Photosystem II 10 kDa polypeptide	6.71	–
	Unigene14296_All	NADPH-quinone oxidoreductase	1.80	−0.25
	Unigene40452_All	Cytochrome b6/f complex subunit VIII	6.04	−5.95
	Unigene5352_All	Ferredoxin-NADP reductase	4.57	−0.32
	Unigene6812_All	Ferredoxin-3	1.92	−0.47
C_4_ carbon fixation	CL2649.Contig3_All	Phosphoenolpyruvate carboxylase	2.14	0.25
	CL6760.Contig2_All	Phosphoenolpyruvate carboxylase	1.44	0.28
	Unigene34895_All	Malate dehydrogenase	5.13	1.01
	CL6022.Contig2_All	Malate dehydrogenase	1.42	−0.16
	CL6958.Contig4_All	NADP-dependent malic enzyme	3.84	1.63
	CL7555.Contig1_All	NAD-dependent malic enzyme	1.57	0.09
	CL791.Contig2_All	Aspartate aminotransferase	1.94	1.58
	CL8006.Contig1_All	Aspartate aminotransferase	1.14	0.47
	CL7761.Contig1_All	Aspartate aminotransferase	1.12	0.25
Chlorophyll biosynthesis	Unigene31043_All	Protoporphyrinogen oxidase	−0.06	1.28
	CL8468.Contig2_All	Protoporphyrinogen oxidase	0.34	7.90
Electron transport	CL4550.Contig3_All	Chlorophyll a-b binding protein	−8.16	1.05
	Unigene173_All	Photosystem II CP43 chlorophyll apoprotein	−0.63	1.03
	Unigene7017_All	Cytochrome f	0.68	1.58
	Unigene10241_All	Photosystem I P700 apoprotein A2	−2.71	1.39
	Unigene18662_All	photosystem I P700 apoprotein A2	−1.74	1.68
	Unigene25839_All	Photosystem I reaction center subunit	–	7.17
Carbon fixation	CL1287.Contig1_All	Malate dehydrogenase [NADP]	−5.27	2.90
	Unigene26311_All	Ribulose 1,5-bisphosphate carboxylase	−5.81	5.73
	Unigene10116_All	Ribulose 1,5-bisphosphate carboxylase	−0.58	1.99
	CL4619.Contig1_All	Alanine aminotransferase 2	−2.15	1.20

### Validation of the DEGs

To further verify the reliability of the transcriptome analysis results, 30 DEGs closely related to this study were randomly selected for qRT-PCR analysis. And all the primers for qPCR were required to amplify single fragments with the expected lengths (100–250 bp, data not shown). The fold changes in relative gene expression measured by qRT-PCR were consistent with the corresponding transcript abundance changes detected by RNA-Seq (Additional file 1:Table S11 and 12). And the linear regression analysis of the fold changes between RNA-Seq and qRT-PCR results exhibited a high correlation, with *R*^2^ = 0.88 and 0.89 in leaves, respectively, under 100 mM NaCl for 6 h (Additional file 1: Figure S4a) and 24 h (Additional file 1: Figure S4b), as well as *R*^2^ = 0.91 and 0.90 in roots, respectively, under 100 mM NaCl for 6 h (Additional file 1: Figure S4c) and 24 h (Additional file 1: Figure S4d), indicating that the RNA-Seq results were highly reliable.

## Discussion

### An efficient ion transport system is a crucial factor in *A. canescens* response to salt

Halophytes depend mostly on the utilization of inorganic ions (such as Na^+^ and K^+^) to maintain shoot osmotic potential and turgor pressure under saline conditions [43]. Ion transport is a crucial factor affecting salt tolerance in plants, playing a fundamental role in homeostasis, signaling and development, particularly in certain halophytes [12, 51]. As a representative secretohalophyte, *A. canescens* possesses unique morphological characteristics (salt bladders) supporting its adaptation to saline environments. Our previous study showed that *A. canescens* could accumulate a great quantity of Na^+^ in leaf tissues as well as salt bladders for OA and could maintain the stability of K^+^ concentration by improving the transport capacity of K^+^ over that of Na^+^ from the stem to leaf under saline conditions, indicating that *A. canescens* could effectively regulate Na^+^/K^+^ homeostasis [40]. Thus, the transcripts of genes related to ion transport is most likely to play an important role in the adaptation of this species to saline conditions.

Leaves are the main organs of salt accumulation for the secretohalophyte *A. canescens*, which is capable of depositing large quantities of salt in EBCs on the leaf surface [40]. How this plant transports and sequesters Na^+^ into EBCs is not clear. The plasma membrane Na^+^/H^+^ antiporter SOS1 mediates cellular Na^+^ efflux [64]. Previous studies found that salt treatment could induce the preferential expression of *AtSOS1* at the root tip, excluding Na^+^ from the root and thereby reducing the absorption of Na^+^ [46]. The high-affinity K^+^ transporter HKT1 mediates cellular Na^+^ influx, and HKT1 in Arabidopsis has been demonstrated to unload Na^+^ from xylem vessels to parenchyma cells and/or control the retrieval of Na^+^ from the xylem to reduce the amount of Na^+^ reaching shoots [9, 49]. Moreover, SOS1 and HKT1 mediate the opposite Na^+^ fluxes under salt treatment to synergistically regulate Na^+^ transport and homeostasis [31]. In addition, the tonoplast Na^+^/H^+^ antiporter NHX has been proven to compartmentalize Na^+^ into vacuoles, and overexpression of NHX could reduce the cytosolic Na^+^ concentration and improve salt tolerance by efficient sequestration [42]. In this study, the transcript levels of *AcSOS1* (CL5512.Contig2_All), *AcHKT1* (CL4724.Contig2_All and CL539.Contig1_All) and *AcNHX* (Unigene2571_All) in leaves were significantly higher under 100 mM NaCl than in the control (Figs. 2 and 3; Additional file 1: Tables S3 and S4), excitedly, *SOS1* and *HKT1* in *A. canescens* were different from in other plants located in roots [9, 47]. Therefore, the excess Na^+^ in the leaves of *A. canescens* is likely to secrete into EBCs via these proteins, that is, Na^+^ is excluded from leaf ECs, probably by SOS1; passed by stalk cells (SCs); and then loaded into EBCs likely by HKT1; finally, Na^+^ may be sequestered in vacuoles by NHX. Recent research has proved that *HKT1;2* expressed in leaves and the EBCs of *C. quinoa* mediates inward Na^+^ currents, which is responsible for loading the Na^+^ into the EBCs [4], strongly supporting our speculation that the *AcHKT1* expressed in leaves directly participated in Na^+^ accumulation in EBCs under saline conditions. In addition, when these proteins transport Na^+^ from ECs into EBCs, plasma- and tonoplast-based H^+^ pumps are required to generate an H^+^ electrochemical gradient [31, 65]. In our study, 100 mM NaCl significantly induced the transcripts of 8 *P-H*^*+*^*-ATPases* and 1 *V-H*^*+*^*-PPase* in leaves (Fig. 3), which might provide a proton pump for secondary transmembrane active transport. In addition, 2 GLUTs in the leaves were upregulated by NaCl, which might unidirectionally move glucose from the mesophyll into EBCs to fuel the above-mentioned H^+^ pumps, and 3 HBs probably facilitated oxygen diffusion into SCs/EBCs to be used for oxidative phosphorylation and ATP production (Fig. 3) [65]. Our data indicate that there is an efficient mechanism in the leaves of *A. canescens* for transporting and sequestering Na^+^, and most importantly, maintaining ion homeostasis under saline conditions by regulating the transcripts of important genes involved in Na^+^ transport.

The accumulation and secretion of a large amount of Na^+^ in leaves facilitate the absorption and transport of Na^+^. Some proteins have been proven to be involved in Na^+^ uptake. A Na^+^/Ca^2+^ exchanger-like protein (NCX, also named NCL) from Arabidopsis probably mediates Na^+^ uptake, since the content of total Na^+^ in *atncl* mutants was obviously lower than that in wild-type (WT) seedlings under salt stress [54]. The cyclic nucleotide-gated channels (CNGCs) may also participate in Na^+^ uptake in Arabidopsis; *AtCNGC3* and *AtCNGC10* were preferentially expressed in the epidermal and endodermal cells in roots, and the knockout of these two genes, respectively, decreased the net Na^+^ influx in roots under salt stress [20, 23]. In *A. canescens*, 1 *NCX* and 4 *CNGCs* were significantly induced by 100 mM NaCl, especially the transcript level of 1 *CNGC* (Unigene12729_All, highly homologous to *CNGC4*), which was significantly increased under 100 mM NaCl for both 6 and 24 h but not under control conditions (Fig. 4; Additional file 1: Tables S5 and S6). These genes might provide an effective pathway for Na^+^ uptake in *A. canescens* under NaCl treatment. After entering the endodermis, Na^+^ would be loaded into the xylem and then transported to the shoot with the transpiration stream. The plasma membrane Na^+^/H^+^ antiporter SOS1 controls long-distance Na^+^ transport [47], and *Zygophyllum xanthoxylum ZxSOS1*-silenced plants exhibit inhibition of growth due to accumulating more Na^+^ in roots but less Na^+^ in leaves and stems than WT plants under salt treatment [33]. The present study found that the transcript abundance of the gene encoding AcSOS1 (CL5512.Contig2_All) in roots was significantly increased under 100 mM NaCl for 24 h (Fig. 4b; Additional file 1: Table S6), indicating that this gene might participate in the long-distance transport of Na^+^ from the roots to shoots in *A. canescens.*

Our previous study showed that *A. canescens* could maintain intracellular K^+^ homeostasis under salt treatment [40]. Numerous studies have identified a series of proteins involved in K^+^ uptake and transport. The inwardly rectifying K^+^ channel AKT1 was not only correlated with K^+^ uptake in roots but also related to Na^+^ homeostasis under saline conditions, and overexpression of *AKT1* improved salt tolerance of transgenic plants by increasing tissue levels of K^+^ [32]. Moreover, the stelar K^+^ outwardly rectifying channel (SKOR) mediates long-distance K^+^ transport and plays an important role in K^+^ accumulation and homeostasis under salt stress [29]. The knockout mutant *atskor* exhibited both lower shoot K^+^ content and lower xylem sap K^+^ concentration compared to WT plants, indicating that SKOR participated in K^+^ release into the xylem sap toward the shoots [16]. The high-affinity K^+^ transporter HAK5 in Arabidopsis participates in K^+^ acquisition and translocation from roots to shoots, and its expression is remarkably upregulated under both K^+^ starvation and salt stress conditions, playing key roles in OA by maintaining K^+^ homeostasis in stress responses [60]. Furthermore, CNGC proteins also mediate K^+^ uptake and transport, and both *atcngc3* and *atcngc10* mutants exhibited lower K^+^ accumulation than WT under salt stress [20, 23]. In *A. canescens*, the transcripts levels of many DEGs related to K^+^ transport were upregulated (Figs. 2 and 4; Additional file 1: Tables S3-S6); in particular, 8 and 9 genes were expressed in the roots and leaves under 100 mM NaCl but not under control conditions for either 6 or 24 h, respectively (Table 2). Among these 8 genes in the roots, 2 *AKT1s*, 2 *CNGCs* and 1 *HAK* might be involved in K^+^ acquisition in roots; 1 *SKOR* gene might mediate long-distance transport of K^+^ from the root to shoot in *A. canescens*. The 9 genes in leaves still included 1 *KEA* and 1 *KCO* in addition to the *AKT*, *SKOR* and *CNGC* families. The K^+^ efflux antiporters (KEAs) are mainly located in plastid-containing organisms and related to chloroplast development and regulation of pH homeostasis, playing an important role in the regulation of monovalent cation efflux to maintain ion homeostasis in plant cells [1]. The two-pore K^+^ channel KCO (also named TPK) localized in the tonoplast is beneficial for maintaining intracellular K^+^ homeostasis by regulating vacuolar K^+^ concentration [19]. Therefore, *A. canescens* possesses an efficient molecular mechanism for modulating K^+^ homeostasis in response to saline conditions.

It is necessary for higher plants to regulate the balance of various nutrients, in addition to Na^+^/K^*+*^ homeostasis, in saline environments. In this study, we found that 100 mM NaCl significantly induced the transcripts of genes encoding proteins that participate in the uptake and transport of important nutrient elements, such as Ca^2+^ (CNGC, cation/Ca^2+^ exchanger CCX and plasma membrane Ca^2+^-ATPase), Mg^2+^ (magnesium transporter MGT), NH_4_^+^ (ammonium transporter AMT), NO^3−^ (nitrate transporter NRT), PO_4_^3−^ (phosphate transporter PHT) and SO_4_^2−^ (sulfate transporter STAS) (Figs. 2 and 4; Additional file 1: Tables S3-S6). Notably, the transcript levels of genes encoding proteins involved in the transport of micronutrient elements were also upregulated in *A. canescens*, and they are mainly involved in the transport of Cu^2+^ (copper transporter CTR), BO^3−^ (boron transporter BOR), Zn^3+^ (zinc transporter ZnT), and MoO_4_^2−^ (molybdate transporter MOT) (Figs. 2 and 4; Additional file 1: Tables S3-S6). Therefore, *A. canescens* could enhance the absorption and transport of nutrient elements by increasing the transcripts of genes encoding transporters/channels for important macro- and microelements to regulate the balance of various nutrients.

### Organic osmolyte accumulation is indispensable for *A. canescens* adaptation to salinity

Under saline conditions, the production of organic osmoprotectants is an important aspect of salt tolerance for higher plants. Our physiological studies have shown that the accumulation of a large amount of compatible solutes (including proline, glycine betaine and soluble sugar) in leaves is an important mechanism of adaptation to saline conditions for *A. canescens* [40]; Guo H. and Bao A.K., unpublished data). Studies have shown that Δ1-pyrroline-5-carboxylate synthetase (P5CS) is a key rate-limiting enzyme in the pathway for proline biosynthesis, and P5CS genes in Arabidopsis play crucial roles in stress regulation and developmental control of proline biosynthesis; *atp5cs1* mutants showed a decrease in stress-induced proline synthesis, resulting in high sensitivity to salt stress and accumulation of reactive oxygen species [50, 58]. Moreover, glutamate (Glu) synthase (GOGAT) plays an important role in the synthesis of Glu, which is an important precursor of proline synthesis and is strongly correlated with proline accumulation [35]. Betaine aldehyde dehydrogenase (BADH) and choline monooxygenase (CMO) are the most critical enzymes catalyzing the synthesis of glycine betaine, and overexpression of the *CMO* and *BADH* genes enhances salt tolerance in transgenic plants [48]. In addition, *BADH* has been characterized in *A. canescens*, and transforming *AcBADH* into soybean improved the salt and drought tolerance of transgenic plants; the higher yield of transgenic lines than of WT plants is particularly exciting [41]. Invertase (INV), sucrose synthase (SuSy) and sucrose phosphate synthase (SPS) are essential enzymes in sucrose metabolism [3, 37]; furthermore, tomato regulates the activity of these enzymes by controlling the expression patterns of their genes to adapt to salinity [30]. Amylase (AMS), mannitol dehydrogenase (MD) and trehalose-6-phosphate synthase (TPS) play indispensable roles in starch, mannitol and trehalose metabolism, respectively, and their enzyme activities are positively related to the salt tolerance of plants [7]. In this study, multiple genes encoding key enzymes for the synthesis of proline, betaine and soluble sugar in leaves were upregulated under 100 mM NaCl (Fig. 5; Additional file 1: Tables S7-S8), and 13 genes (such as *P5CS*, *BADH* and *SPS*) of these upregulated genes were continuously expressed under salt treatment for both 6 and 24 h (Table 3), suggesting that *A. canescens* possesses an efficient mechanism for accumulating osmoprotectants under saline conditions by modulating the expression patterns of important genes involved in compatible solute biosynthesis, which would be conducive to protecting plants in saline environments by OA.

### Aquaporin plays an important role in the regulation of water balance in *A. canescens* under saline conditions

*A. canescens* seedlings can maintain a higher leaf relative water content by effective OA under salinity treatment [40]. Moreover, our latest study found that water was abundantly transported as a solvent into salt bladders with the accumulation of Na^+^ in salt bladders of *A. canescens* under NaCl treatment, causing rapid expansion of the salt bladders, a sharp increase in turgor pressure and eventually the bursting of the bladders and subsequent release of a large amount of accumulated Na^+^ (Guo H. and Bao A.K., unpublished data); this finding suggests that the rapid accumulation of water in salt bladders is the key factor affecting salt secretion in *A. canescens*. AQPs can effectively regulate the water balance inside and outside of the plant cell by specifically mediating the rapid transmembrane transport of water [6]. In this study, many DEGs related to AQPs were upregulated in leaves but not in roots under 100 mM NaCl for 6 h, including 6 nodulin-like intrinsic proteins (NIPs), 1 small basic intrinsic protein (SIP), 2 plasma membrane intrinsic proteins (PIPs) and 3 tonoplast intrinsic proteins (TIPs) (Table 4). The NIPs mainly mediate boron uptake (NIP5;1) or are involved in pollen development and pollination (NIP4) in *Arabidopsis thaliana* [10, 55]*.* The SIPs are localized to the endoplasmic reticulum membrane but currently are not well characterized [25]. Plasma membrane-located PIPs are divided into the PIP1 and PIP2 groups; PIP2 members mainly function as water channels, while PIP1 members usually have much lower or no water conductivity due to their failure to localize to the plasma membrane [5]. Our results showed that the transcript of 1 *PIP* gene (CL969.Contig2_All, highly homologous to *PIP2;3*) was significantly upregulated in leaves (Table 4). PIP2 in *Malus zumi* Mats is involved in water movement during both water absorption and transport and alters the salt tolerance of transgenic Arabidopsis [53]. An AQP protein, AcPIP2, characterized from *A. canescens*, improved plant growth rate and salt tolerance when overexpressed in *A. thaliana* [28]. Moreover, tonoplast-located TIPs, primarily mediating the accumulation of water in the vacuole, play vital roles in maintaining cell turgor and enhancing the capacity for OA and are also able to indirectly promote Na^+^ compartmentation into vacuoles, which is conducive to improving plant adaptation to saline environments [25, 36]. In the present study, we found that the transcripts of 3 *TIPs* (Unigene15728_All, highly homologous to *TIP2;2*; Unigene12348_All, highly homologous to *TIP4;1* and CL5261.Contig2_All, highly homologous to *TIP1;3*) were sharply upregulated in the leaves but downregulated in the roots of *A. canescens* under NaCl treatment, and there was even a 14.24-fold increase in the transcript level of *AcTIP2;2* (Table 4). Therefore, the AcPIP2 and AcTIPs in the leaves of *A. canescens* are likely to be involved in the transport of water into salt bladders under salt treatment, which facilitates salt secretion and the maintenance of the water balance in leaves and might result from the accumulation of solutes in leaf tissues and salt bladders.

### Moderate salinity improves photosynthesis of *A. canescens* by increasing the transcripts of photosynthesis-related genes

Previous studies showed that NaCl significantly improved the photosynthetic capacity of *A. canescens* plants, and the trends of photosynthetic indicators were different from those of C_3_ xerohalophytes, suggesting that Na^+^ may promote the C_4_ photosynthetic process of *A. canescens* under saline conditions [40]. C_4_ plants share stronger CO_2_ assimilation capacity and can sufficiently utilize light energy; moreover, this kind of plant have greater adaptability to adversity since they can take advantage of low CO_2_ levels in the intercellular space under stress conditions [15, 27]. The oxygenic photosynthesis of higher plants can be divided into three stages: the primary reaction, photosynthetic electron transport and photophosphorylation and CO_2_ assimilation. The first two steps in this process involve the conversion of sunlight into active chemical energy, which is driven by several multisubunit membrane protein complexes, including photosystem II, cytochrome *b6/f*, photosystem I, ferredoxin and ATP synthase [38]. The last reaction is a process that converts CO_2_ into stable chemical energy stored in organic matter by using the energy (ATP and NADPH) produced by the light reaction [15]. Our study found that the majority of DEGs related to the above-mentioned complexes and the enzymes involved in carbon fixation and chlorophyll biosynthesis/catabolism were downregulated under 100 mM NaCl for 6 h (Fig. 7a, Additional file 1: S13), but the few upregulated genes were mainly involved in electron transport and carbon fixation, the latter of which were almost always involved in the C_4_ pathway, including phosphoenolpyruvate carboxylase (PEPC), malate dehydrogenase (MDH), malic enzyme (ME) and aspartate aminotransferase (AST) (Table 5). These enzymes play key roles in the C_4_ photosynthetic pathway and are capable of providing more CO_2_ for the C_3_ pathway in the vascular bundle sheath^61^. Interestingly, the transcript levels of many DEGs involved in the processes of chlorophyll biosynthesis, electron transport and carbon fixation (based on the C_3_ pathway) were significantly upregulated after treatment with 100 mM NaCl for 24 h but not after treatment for 6 h (Table 5), suggesting that *A. canescens* preferentially increased the transcript abundances of genes encoding key enzymes in the C_4_ pathway to improve its assimilation capacity and then increased the transcript levels of other genes encoding complexes related to chlorophyll biosynthesis, electron transport and C_3_ carbon fixation under salt treatment, which might be one of the important reasons the photosynthesis of *A. canescens* was significantly improved by 100 mM NaCl [40]. At present, re-engineering C_3_ plants with C_4_ CO_2_-concentrating mechanisms is of broad interest [34, 45]. Overexpression of plastidic *ZmNADP-MDH* (NADP-MDH) in maize conferred salt tolerance to transgenic Arabidopsis [24]*.* Thus, the results of this study provide abundant genetic resources for improving photosynthetic efficiency in C_3_ crops/forages through genetic engineering.

## Conclusions

This study identified candidate genes showing significant transcriptional changes in *A. canescens* under 100 mM NaCl treatment, mainly focusing on genes related to ion transport, organic osmolyte synthesis, water transport and photosynthesis. The abundance of transcripts encoding transporters/channels for important macro- and microelements was significantly increased by 100 mM NaCl, which is conducive to promote the uptake and transport of nutrient elements. It is worth noting that some genes related to Na^+^ transport in leaves (such as *AcSOS1*, *AcHKT1* and *AcNHX*) might play crucial roles in the excretion of salt via epidermis bladder cells. In addition, the transcripts of a number of genes related to the synthesis of organic osmolytes in leaves was significantly upregulated by NaCl treatment, which allowed the accumulation of more organic solutes to enhance OA under salt treatment. Moreover, 100 mM NaCl promoted water transport in *A. canescens* by inducing the transcripts of aquaporin-encoding genes in leaves. Interestingly, NaCl preferentially induced the transcripts of genes encoding proteins participating in the C_4_ photosynthetic pathway to provide greater assimilation capacity for photosynthesis and then increased the transcript levels of other genes encoding complexes related to chlorophyll biosynthesis, electron transport and C_3_ carbon fixation under salt treatment. Our results lay the foundation for investigating molecular mechanisms of salt tolerance in secretohalophytes and provide a theoretical basis for genetic improvement of stress tolerance in important crops and forages by using the outstanding gene resources from *A. canescens*.

## Methods

### Plant materials and experimental treatments

Seeds of *Atriplex canescens* were collected from Lingwu County in Ningxia Autonomous Region, China. After removed the hard seed coat with 75% H_2_SO_4_ (v/v) for 15 h, the seeds were washed many times with purified water until the washings has no smell and then germinated in moist vermiculite at 28 °C in the dark for 5 days. Uniform seedlings were transplanted into plastic containers (5 cm × 5 cm × 5 cm; two plants/pot) filled with vermiculite and irrigated with 1/2-strength Hoagland nutrient solution at 2-day intervals [40]. Plants were cultured at 28 °C/25 °C (day/night), 16/8 h photoperiod (light/dark; the light density was approximately 800 μmol/m^2^/s) and 65% relative humidity.

Four-week-old seedlings were treated with 1/2-strength Hoagland nutrient solution supplemented with 0 (control) or 100 mM NaCl. The leaves and roots of seedlings in the two treatments were collected after treatment for 6 and 24 h, respectively. A total of eight samples were marked as follows: CL6, CR6, SL6, SR6, CL24, CR24, SL24 and SR24; C and S represent the control and treatment with 100 mM NaCl, respectively; 6 and 24 denote the treatment duration; and R and L denote the roots and leaves, respectively. For example, SL6 and SR6 were the leaf and root samples, respectively, from salt-treated plants for which seedlings were treated for 6 h. All the fresh samples were immediately frozen in liquid nitrogen and stored at − 80 °C until RNA extraction.

### RNA preparation, cDNA library construction and Illumina sequencing

Total RNA was isolated from the eight samples with an RNeasy Plant Mini Kit (Qiagen). The extracted RNA was quantified by using a NanoDrop ND-1000 instrument (Thermo Scientific), and the integrity of the RNA was determined by 1% agarose gel electrophoresis. Equivalent amounts of total RNA isolated from each of the four leaf tissues (CL6, SL6, CL24 and SL24) and each of the four corresponding root tissues (CR6, SR6, CR24 and SR24) were pooled. The two mRNA pools were then used for reverse transcription to obtain two cDNA libraries as the cDNA in the leaves and roots of *A. canescens* by using the method described by Dang et al. [8] and sequenced on an Illumina HiSeq™ 2000 platform in BGI Shenzhen.

### De novo assembly and functional annotation

High-quality clean reads were created after filtering adaptor sequences, duplicated sequences, reads containing more than 5% ambiguous bases (‘N’) and reads in which more than 50% of bases showed a *Q*-value ≤5. After filtering out low quality reads, de novo assembly was proceeded by using Trinity; then, the Trinity unigenes were clustered with TGICL software to minimize sequence redundancy [21]. The unigenes were divided into two classes after performing gene family clustering, one class included clusters with the prefix CL contained several unigenes with a sequence similarity of more than 70%, and the other included singletons with the prefix unigenes [31]. To attach predicted gene informations for assembled unigenes, the sequences were functionally annotated based on seven protein databases (including the Nr, Nt, Swiss-Prot, Kyoto Encyclopedia of Genes and Genomes (KEGG), Clusters of Orthologous Groups (COG), InterPro and Gene Ontology (GO) databases) using the BLASTX tool with an *E*-value ≤10^− 5^ threshold. Blast2GO software was employed to perform functional categorization by GO terms on the basis of biological process, cellular component and molecular function ontologies, and the Web Gene Ontology Annotation Plot (WEGO) tool was used to statistically analyze the data [8].

### Differentially expressed gene (DEG) library preparation and analysis

Eight independent cDNA libraries (CL6, CR6, SL6, SR6, CL24, CR24, SL24 and SR24) were prepared in parallel for leaves and roots at different times under salt treatment by using a tag-based DGE kit [59]. Then, each library was sequenced through the Illumina HiSeq™ 2000 sequencing platform in BGI Shenzhen. After low-quality reads (including reads with adaptors, more than 10% unknown nucleotides (‘N’) and only one copy number) were removed, the clean reads were mapped to the transcriptome reference database. And then, the transcript levels of all assembled unigenes were calculated by using the number of fragments per kb per million reads (FPKM) method to identify differentially expressed genes (DEGs) [59]. In addition, the false discovery rate (FDR) method was used to confirm the threshold *P*-value for multiple tests and analysis by manipulating the FDR value. An FDR < 0.001 and an absolute value of |log_2_Ratio| > 1 were used as thresholds to identify DEGs [8].

### qRT-PCR validation of DEGs

To experimentally evaluate the RNA-Seq results, total RNA was extracted from the 8 samples as described before and reversely transcribed into cDNA according to the manufacturer’s protocol (TaKaRa Biotechnology). The qRT-PCR was conducted by using SYBR Green Real-Time PCR Master Mix (TaKaRa Biotechnology) and performed on a StepOnePlus Real-Time PCR Thermocycler (Applied Biosystems, USA). *ACTIN* gene was used as the internal standard. The relative transcript levels of the 30 randomly selected unigenes were calculated using the 2^-ΔΔCt^ method [31].

## Additional file

Additional file 1 : **Figure S1.** Length distribution of all assembled unigenes. **Figure S2.** COG function distribution of all unigenes. A total of 26,012 putative proteins showing significant homology to those in the COG database were classified into 25 functional clusters. X-axis indicates the number of unigenes in a cluster. **Figure S3.** GO function distribution of all unigenes. A total of 37,395 unigenes were assigned to GO terms and were summarized in 3 main GO categories and 52 subcategories. X-axis indicates the number of genes in a category. **Figure S4.** Correlation analysis for expression pattern validation of 30 randomly selected DEGs between RNA-Seq and qRT-PCR results. **Table S1.** Summary of sequencing reads after filtering. **Table S2.** Summary of sequence annotation. **Table S3.** Differentially expressed genes (DEGs) related to ion transport in leaves of *A. canescens* under 100 mM NaCl for 6 h. **Table S4.** DEGs related to ion transport in leaves of *A. canescens* under 100 mM NaCl for 24 h. **Table S5.** DEGs related to ion transport in roots of *A. canescens* under 100 mM NaCl for 6 h. **Table S6.** DEGs related to ion transport in roots of *A. canescens* under 100 mM NaCl for 24 h. **Table S7.** DEGs related to organic osmolytes synthesis in leaves of *A. canescens* under 100 mM NaCl for 6 h. **Table S8.** DEGs related to organic osmolytes synthesis in leaves of *A. canescens* under 100 mM NaCl for 24 h. **Table S9.** DEGs related to photosynthesis in leaves of *A. canescens* under 100 mM NaCl for 6 h. **Table S10.** DEGs related to photosynthesis in leaves of *A. canescens* under 100 mM NaCl for 24 h. **Table S11.** Expression pattern validation of 30 randomly selected genes in leaves of *A. canescens* under 100 mM NaCl for 6 and 24 h by qRT-PCR. **Table S12.** Expression pattern validation of 30 randomly selected genes in roots of *A. canescens* under 100 mM NaCl for 6 and 24 h by qRT-PCR. (DOCX 1081 kb)

## Abbreviations

AKT: Inwardly rectifying K^+^ channel
AMS: Amylase
AMT: NH_4_^+^ transporter
BADH: Betaine aldehyde dehydrogenase
BOR: BO^3−^ transporter
CCX: Cation/Ca^2+^ exchanger
CLC: Vacuolar Cl^−^/H^+^ exchanger
CMO: Choline monooxygenase
CNGC: Cyclic nucleotide-gated channel
COG: Clusters of orthologous groups
CTR: Cu^2+^ transporter
DEG: Differentially expressed gene
EBC: Epidermal bladder cell
EC: Epidermal cell
FDR: False discovery rate
FPKM: Number of transcripts per million clean reads
GDH: Glutamate dehydrogenase
GLUT: Glucose transporter
GO: Gene ontology
GOGAT: Gluamate synthase
HB: Hemoglobin
HKT: High-affinity K^+^ transporter
INV: Invertase
KEGG: Kyoto encyclopedia of genes and genomes
KT/HAK/KUP: K^+^ transporter
MD: Mannitol dehydrogenase
MGT: Mg^2+^ transporter
MOT: MoO_4_^2−^ transporter
NHX: Tonoplast Na^+^/H^+^ antiporter
NIP: Nnodulin-like intrinsic protein
NRT: NO_3_^−^ transpoter
OAT: Ornithine aminotransferase
P5CS: Δ1-pyrroline-5-carboxylate synthetase
P-Ca^2+^ ATPase: Plasma membrane Ca^2+^ ATPase
PEAMT: Phosphoethanolamine N-methyltransferase
P-H^+^ ATPase: Plasma membrane H^+^ ATPase
PHT: PO_4_^3−^ transporter
PIP: Plasma membrane intrinsic protein
SC: Stalk cell
SIP: Small basic intrinsic protein
SKOR: Stelar K^+^ outward rectifying channel
SLAH: Slow type anion channel
SOS1: Plasma membrane Na^+^/H^+^ antiporter
SPS: Sucrose-phosphate synthase
SS: Starch synthase
STAS: SO_4_^2−^ transporter
SuSy: Sucrose synthase
TIP: Tonoplast intrinsic protein
TPS: Trehalose-phosphate synthase
V-CAX: Vacuolar cation/H^+^ exchanger
V-H^+^ PPase: Vacuolar H^+^ PPase
ZnT: Zn^3+^ transporter
