# Supplementary material for: Identification of candidate genes related to salt tolerance of the secretohalophyte Atriplex canescens by transcriptomic analysis
Source: BMC Plant Biol. 2019 May 22;19:213. doi: 10.1186/s12870-019-1827-6 (PMC6532215; doi:10.1186/s12870-019-1827-6)
Supplement: Supplementary file 1 — : Figure S1. Length distribution of all assembled unigenes. Figure S2. COG function distribution of all unigenes. A total of 26,012 putative proteins showing significant homology to those in the COG database were classified into 25 functional clusters. X-axis indicates the number of unigenes in a cluster. Figure S3. GO function distribution of all unigenes. A total of 37,395 unigenes were assigned to GO terms and were summarized in 3 main GO categories and 52 subcategories. X-axis indicates the number of genes in a category. Figure S4. Correlation analysis for expression pattern validation of 30 randomly selected DEGs between RNA-Seq and qRT-PCR results. Table S1. Summary of sequencing reads after filtering. Table S2. Summary of sequence annotation. Table S3. Differentially expressed genes (DEGs) related to ion transport in leaves of A. canescens under 100 mM NaCl for 6 h. Table S4. DEGs related to ion transport in leaves of A. canescens under 100 mM NaCl for 24 h. Table S5. DEGs related to ion transport in roots of A. canescens under 100 mM NaCl for 6 h. Table S6. DEGs related to ion transport in roots of A. canescens under 100 mM NaCl for 24 h. Table S7. DEGs related to organic osmolytes synthesis in leaves of A. canescens under 100 mM NaCl for 6 h. Table S8. DEGs related to organic osmolytes synthesis in leaves of A. canescens under 100 mM NaCl for 24 h. Table S9. DEGs related to photosynthesis in leaves of A. canescens under 100 mM NaCl for 6 h. Table S10. DEGs related to photosynthesis in leaves of A. canescens under 100 mM NaCl for 24 h. Table S11. Expression pattern validation of 30 randomly selected genes in leaves of A. canescens under 100 mM NaCl for 6 and 24 h by qRT-PCR. Table S12. Expression pattern validation of 30 randomly selected genes in roots of A. canescens under 100 mM NaCl for 6 and 24 h by qRT-PCR. (DOCX 1081 kb) [file 12870_2019_1827_MOESM1_ESM.docx]

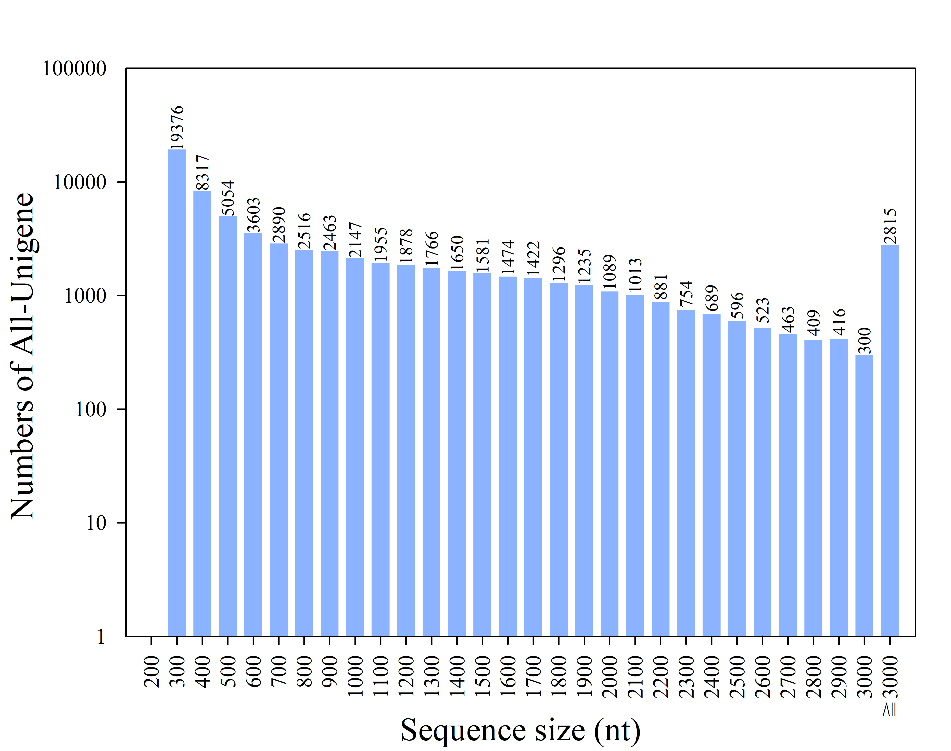


**Figure S1** Length distribution of all assembled unigenes.

**Figure S2** COG function distribution of all unigenes. A total of 26012 putative proteins showing significant homology to those in the COG database were classified into 25 functional clusters. X-axis indicates the number of unigenes in a cluster.

**Figure S3** GO function distribution of all unigenes. A total of 37395 unigenes were assigned to GO terms and were summarized in 3 main GO categories and 52 subcategories. X-axis indicates the number of genes in a category.

**Figure S4** Correlation analysis for expression pattern validation of 30 randomly selected DEGs between RNA-Seq and qRT-PCR results. The linear regression analysis of the transcript abundance of these genes between RNA-Seq and qRT-PCR data in leaves of *A. canescens* under 100 mM NaCl for 6 h (a) and 24 h (b), and in roots under 100 mM NaCl for 6 h (c) and 24 h (d).

**Table** S1 Summary of sequencing reads after filtering.

| Samples | Total Clean Bases (Gb) | Total Raw Reads (Mb) | Total Clean Reads (Mb) | Clean Reads  Q20 (%) | N percentage (%) |
| --- | --- | --- | --- | --- | --- |
| Leaves | 13.37 | 207.20 | 133.70 | 97.89 | 0.00 |
| Roots | 13.41 | 210.00 | 134.12 | 97.95 | 0.00 |

**Table** S2 Summary of sequence annotation.

| Values | Nr | Nt | Swiss-Prot | KEGG | COG | Interpro | GO | Overall |
| --- | --- | --- | --- | --- | --- | --- | --- | --- |
| Number | 39,001 | 32,331 | 26,561 | 29,321 | 15,970 | 29,911 | 7,778 | 44,121 |
| Percentage | 55.26% | 45.81% | 37.64% | 41.55% | 22.63% | 42.38% | 11.02% | 62.52% |

**Table S3** Differentially expressed genes (DEGs) related to ion transport in leaves of *A. canescens* under 100 mM NaCl for 6 h. CL6 indicates the leaves of plants treated as a control for 6 h, SL6 indicates leaves of plants treated with 100 mM NaCl for 6 h, and “Fold change” equals log_2_ (FPKM-SL6/FPKM-CL6).

| **Gene ID** | **FPKM-CL6** | **FPKM-SL6** | **Fold change** | **Homologous species** |
| --- | --- | --- | --- | --- |
| **NHX** | | | | |
| Unigene2571_All | 3.96 | 14.26 | 1.85 | *Mesembryanthemum crystallinum* |
| Unigene2433_All | 64.36 | 28.28 | -1.19 | *Atriplex halimus* |
| **HKT** | | | | |
| CL539.Contig1_All | 0.10 | 15.46 | 7.27 | *Nelumbo nucifera* |
| **SKOR** | | | | |
| CL3066.Contig1_All | 0.67 | 25.24 | 5.24 | *Alternanthera philoxeroides* |
| Unigene39479_All | 4.10 | 0.41 | -3.32 | *Alternanthera philoxeroides* |
| **KEA** | | | | |
| Unigene14970_All | 4.59 | 14.35 | 1.64 | *Nicotiana tomentosiformis* |
| CL3380.Contig1_All | 54.15 | 1.61 | -5.07 | *Nelumbo nucifera* |
| **KCO** | | | | |
| CL6946.Contig2_All | 0.32 | 1.88 | 2.55 | *Vitis vinifera* |
| **AKT** | | | | |
| Unigene25880_All | 0.01 | 19.42 | 10.92 | *Mesembryanthemum crystallinum* |
| CL6985.Contig2_All | 0.01 | 10.57 | 10.05 | *Mesembryanthemum crystallinum* |
| Unigene1023_All | 0.23 | 17.61 | 6.26 | *Mesembryanthemum crystallinum* |
| Unigene11168_All | 0.12 | 2.27 | 4.24 | *Glycine max* |
| Unigene17093_All | 0.19 | 2.97 | 3.97 | *Nicotiana tomentosiformis* |
| CL1419.Contig2_All | 6.06 | 16.43 | 1.44 | *Mesembryanthemum crystallinum* |
| CL5980.Contig3_All | 10.81 | 0.01 | -10.08 | *Vitis vinifera* |
| **KT/HAK/KUP** | | | | |
| CL2081.Contig2_All | 0.01 | 4.48 | 8.81 | *Nelumbo nucifera* |
| Unigene503_All | 14.03 | 2.14 | -2.71 | *Vitis vinifera* |
| CL9787.Contig2_All | 54.27 | 12.38 | -2.13 | *Celosia argentea* |
| **CNGC** | | | | |
| CL7131.Contig1_All | 0.01 | 3.02 | 8.24 | *Alternanthera philoxeroides* |
| CL1899.Contig3_All | 1.95 | 14.07 | 2.85 | *Theobroma cacao* |
| CL2537.Contig1_All | 4.49 | 14.99 | 1.74 | *Solanum lycopersicum* |
| Unigene28871_All | 0.22 | 0.65 | 1.56 | *Nicotiana tabacum* |
| Unigene5732_All | 0.75 | 1.68 | 1.16 | *Theobroma cacao* |
| Unigene16063_All | 5.50 | 11.12 | 1.02 | *Vitis vinifera* |
| CL5475.Contig2_All | 2.25 | 1.01 | -1.16 | *Nelumbo nucifera* |
| Unigene142_All | 2.07 | 0.48 | -2.11 | *Cicer arietinum* |
| Unigene12729_All | 7.63 | 0.01 | -9.58 | *Ricinus communis* |
| **CCX** | | | | |
| CL5759.Contig2_All | 4.58 | 19.92 | 2.12 | *Vitis vinifera* |
| CL1695.Contig1_All | 2.56 | 6.40 | 1.32 | *Vitis vinifera* |
| **P-Ca^2+^ ATPase** | | | | |
| Unigene9206_All | 0.42 | 12.97 | 4.95 | *Vitis vinifera* |
| Unigene10043_All | 2.13 | 29.15 | 3.77 | *Sesuvium portulacastrum* |
| CL1941.Contig1_All | 17.78 | 66.03 | 1.89 | *Vitis vinifera* |
| Unigene5657_All | 1.18 | 2.60 | 1.14 | *Theobroma cacao* |
| CL4700.Contig2_All | 3.83 | 1.68 | -1.19 | *Vitis vinifera* |
| **V-CAX** | | | | |
| Unigene12604_All | 2.01 | 7.25 | 1.85 | *Sesuvium portulacastrum* |
| CL8183.Contig1_All | 8.09 | 23.59 | 1.54 | *Sesuvium portulacastrum* |
| **P-H^+^ ATPase** | | | | |
| Unigene14649_All | 13.43 | 83.86 | 2.64 | *Sesuvium portulacastrum* |
| Unigene9556_All | 70.76 | 402.3 | 2.51 | *Sesuvium portulacastrum* |
| Unigene447_All | 4.09 | 20.69 | 2.34 | *Solanum tuberosum* |
| CL8803.Contig1_All | 0.82 | 3.93 | 2.26 | *Sesuvium portulacastrum* |
| CL6839.Contig2_All | 9.27 | 40.89 | 2.14 | *Sesuvium portulacastrum* |
| CL6839.Contig1_All | 73.66 | 33.77 | -1.13 | *Sesuvium portulacastrum* |
| CL6971.Contig1_All | 79.21 | 22.87 | -1.79 | *Mesembryanthemum crystallinum* |
| **V-H^+^ PPase** | | | | |
| CL4556.Contig4_All | 5.76 | 39.12 | 2.76 | *Salicornia europaea* |
| **MGT** | | | | |
| CL7136.Contig1_All | 0.35 | 2.75 | 2.97 | *Suaeda glauca* |
| CL9565.Contig2_All | 1.26 | 6.10 | 2.28 | *Cucumis sativus* |
| Unigene4950_All | 16.99 | 8.37 | -1.02 | *Vitis vinifera* |
| CL7136.Contig4_All | 3.63 | 0.01 | -8.50 | *Vitis vinifera* |
| **AMT** | | | | |
| Unigene1739_All | 1.07 | 10.76 | 3.33 | *Eucalyptus grandis* |
| CL89.Contig3_All | 6.79 | 16.94 | 1.32 | *Coffea canephora* |
| Unigene1346_All | 7.20 | 0.01 | -9.49 | *Alternanthera philoxeroides* |
| Unigene10940_All | 2.92 | 0.01 | -8.19 | *Medicago truncatula* |
| **NRT** | | | | |
| CL8745.Contig1_All | 0.01 | 50.29 | 12.30 | *Ricinus communis* |
| Unigene32523_All | 0.01 | 2.38 | 7.89 | *Solanum lycopersicum* |
| Unigene31327_All | 0.01 | 1.83 | 7.52 | *Beta vulgaris* |
| Unigene30382_All | 0.01 | 1.72 | 7.43 | *Spinacia oleracea* |
| Unigene26958_All | 0.01 | 1.32 | 7.04 | *Spinacia oleracea* |
| Unigene6575_All | 0.01 | 1.03 | 6.69 | *Gossypium hirsutum* |
| Unigene26957_All | 0.01 | 0.98 | 6.61 | *Spinacia oleracea* |
| Unigene31057_All | 0.01 | 0.95 | 6.57 | *Arabidopsis thaliana* |
| Unigene36682_All | 0.01 | 0.93 | 6.54 | *Spinacia oleracea* |
| Unigene27723_All | 0.01 | 0.53 | 5.73 | *Ricinus communis* |
| Unigene29763_All | 0.01 | 0.25 | 4.64 | *Spinacia oleracea* |
| CL3698.Contig4_All | 0.10 | 6.60 | 6.04 | *Spinacia oleracea* |
| CL2829.Contig1_All | 0.20 | 5.69 | 4.83 | *Vitis vinifera* |
| CL6484.Contig1_All | 1.31 | 36.28 | 4.79 | *Vitis vinifera* |
| Unigene12841_All | 0.59 | 11.01 | 4.22 | *Citrus sinensis* |
| CL2228.Contig2_All | 1.94 | 12.93 | 2.74 | *Theobroma cacao* |
| CL8524.Contig2_All | 4.45 | 12.08 | 1.44 | *Vitis vinifera* |
| Unigene8885_All | 0.56 | 1.13 | 1.01 | *Morus notabilis* |
| CL6490.Contig1_All | 44.85 | 0.63 | -6.15 | *Vitis vinifera* |
| CL3030.Contig6_All | 5.73 | 0.01 | -9.16 | *Vitis vinifera* |
| **STAS** | | | | |
| CL6653.Contig1_All | 0.05 | 42.15 | 9.72 | *Theobroma cacao* |
| Unigene765_All | 33.68 | 79.83 | 1.25 | *Nicotiana sylvestris* |
| CL2358.Contig1_All | 51.69 | 17.16 | -1.59 | *Populus tremula* |
| CL4513.Contig1_All | 6.99 | 1.88 | -1.89 | *Sesuvium portulacastrum* |
| CL576.Contig1_All | 5.59 | 1.08 | -2.37 | *Camellia sinensis* |
| **PHT** | | | | |
| CL5611.Contig3_All | 0.01 | 97.58 | 13.25 | *Glycine max* |
| Unigene5190_All | 0.01 | 5.57 | 9.12 | *Populus trichocarpa* |
| CL5240.Contig1_All | 0.25 | 31.24 | 6.97 | *Medicago sativa* |
| Unigene5615_All | 0.12 | 6.20 | 5.69 | *Medicago truncatula* |
| Unigene4292_All | 3.75 | 22.98 | 2.62 | *Vitis vinifera* |
| CL7475.Contig1_All | 31.41 | 0.67 | -5.55 | *Spinacia oleracea* |
| **CLC** | | | | |
| CL2875.Contig1_All | 3.16 | 11.36 | 1.85 | *Vitis vinifera* |
| CL1227.Contig3_All | 29.79 | 70.18 | 1.24 | *Vitis vinifera* |
| **SLAH** | | | | |
| Unigene12189_All | 0.07 | 55.01 | 9.62 | *Morus notabilis* |
| Unigene12869_All | 3.10 | 1.19 | -1.38 | *Eucalyptus grandis* |
| **CTR** | | | | |
| Unigene1692_All | 0.01 | 20.03 | 10.97 | *Theobroma cacao* |
| Unigene464_All | 0.01 | 15.21 | 10.57 | *Ricinus communis* |
| Unigene14371_All | 10.89 | 0.01 | -10.09 | *Theobroma cacao* |
| **BOR** | | | | |
| CL2956.Contig3_All | 2.46 | 19.67 | 3.00 | *Theobroma cacao* |
| CL6313.Contig1_All | 7.59 | 44.65 | 2.56 | *Vitis vinifera* |
| Unigene1509_All | 2.11 | 16.49 | 2.97 | *Populus trichocarpa* |
| Unigene4180_All | 6.08 | 32.63 | 2.42 | *Cucumis sativus* |
| **ZnT** | | | | |
| CL9112.Contig1_All | 1.48 | 15.67 | 3.40 | *Medicago truncatula* |
| Unigene6856_All | 3.61 | 25.54 | 2.82 | *Solanum lycopersicum* |
| CL3501.Contig1_All | 2.71 | 9.54 | 1.82 | *Vitis vinifera* |
| Unigene13213_All | 26.17 | 61.53 | 1.23 | *Chenopodium quinoa* |
| Unigene17547_All | 11.04 | 25.51 | 1.21 | *Theobroma cacao* |
| Unigene26807_All | 12.10 | 1.76 | -2.78 | *Nicotiana tabacum* |
| **MOT** | | | | |
| Unigene7283_All | 2.08 | 31.82 | 3.94 | *Nicotiana tomentosiformis* |

**Table S4** DEGs related to ion transport in leaves of *A. canescens* under 100 mM NaCl for 24 h. CL24 indicates the leaves of plants treated as a control for 24 h, SL24 indicates leaves of plants treated with 100 mM NaCl for 24 h, and “Fold change” equals log_2_ (FPKM-SL24/FPKM-CL24).

| **Gene ID** | **FPKM-CL24** | **FPKM-SL24** | **Fold change** | **Homologous species** |
| --- | --- | --- | --- | --- |
| **SOS1** |  |  |  |  |
| CL5512.Contig2_All | 0.01 | 9.34 | 9.87 | *Chenopodium quinoa* |
| **HKT** | | | | |
| CL4724.Contig2_All | 2.28 | 12.83 | 2.49 | *Mesembryanthemum crystallinum* |
| **KEA** | | | | |
| Unigene23223_All | 0.01 | 0.66 | 6.04 | *Nelumbo nucifera* |
| **KCO** | | | | |
| CL6946.Contig2_All | 0.01 | 1.04 | 6.70 | *Vitis vinifera* |
| Unigene7902_All | 3.98 | 1.2 | -1.73 | *Morus notabilis* |
| **SKOR** | | | | |
| Unigene39479_All | 0.01 | 1.77 | 7.47 | *Alternanthera philoxeroides* |
| CL3066.Contig2_All | 0.14 | 0.67 | 2.26 | *Alternanthera philoxeroides* |
| Unigene26985_All | 0.91 | 2.02 | 1.15 | *Alternanthera philoxeroides* |
| **AKT** | | | | |
| Unigene23528_All | 0.01 | 1.18 | 6.88 | *Mesembryanthemum crystallinum* |
| Unigene1023_All | 0.11 | 0.55 | 2.32 | *Mesembryanthemum crystallinum* |
| CL6985.Contig2_All | 0.38 | 1.16 | 1.61 | *Mesembryanthemum crystallinum* |
| Unigene11168_All | 0.40 | 0.18 | -1.15 | *Glycine max* |
| **KT/HAK/KUP** | | | | |
| CL2081.Contig2_All | 0.01 | 0.57 | 5.83 | *Nelumbo nucifera* |
| **CNGC** | | | | |
| CL1899.Contig4_All | 0.01 | 0.60 | 5.91 | *Vitis vinifera* |
| CL5475.Contig2_All | 1.05 | 3.49 | 1.73 | *Nelumbo nucifera* |
| Unigene19313_All | 0.37 | 1.10 | 1.57 | *Vitis vinifera* |
| CL7131.Contig1_All | 0.38 | 1.06 | 1.48 | *Beta vulgaris* |
| CL2537.Contig1_All | 2.54 | 6.26 | 1.30 | *Solanum lycopersicum* |
| CL1686.Contig1_All | 0.89 | 2.14 | 1.27 | *Nicotiana tomentosiformis* |
| CL4135.Contig2_All | 1.48 | 2.99 | 1.01 | *Vitis vinifera* |
| **CCX** | | | | |
| CL5759.Contig1_All | 2.58 | 1.04 | -1.31 | *Arabidopsis thaliana* |
| **P-Ca^2+^ ATPase** | | | | |
| Unigene5657_All | 2.37 | 1.04 | -1.19 | *Vitis vinifera* |
| **V-CAX** | | | | |
| CL8183.Contig3_All | 0.46 | 0.01 | -5.52 | *Sesuvium portulacastrum* |
| **P-H^+^ ATPase** | | | | |
| Unigene40626_All | 1.32 | 6.35 | 2.27 | *Sesuvium portulacastrum* |
| Unigene19179_All | 0.99 | 2.77 | 1.48 | *Populus trichocarpa* |
| CL2639.Contig4_All | 0.07 | 0.01 | -2.81 | *Eucalyptus grandis* |
| **MGT** | | | | |
| CL7136.Contig4_All | 0.01 | 1.72 | 7.43 | *Vitis vinifera* |
| CL8692.Contig1_All | 0.01 | 3.52 | 8.46 | *Populus trichocarpa* |
| CL1495.Contig7_All | 1.69 | 5.34 | 1.66 | *Cucumis sativus* |
| **NRT** | | | | |
| CL3698.Contig3_All | 0.01 | 0.96 | 6.58 | *Spinacia oleracea* |
| Unigene4441_All | 0.01 | 0.64 | 6.00 | *Solanum tuberosum* |
| Unigene12841_All | 0.01 | 0.61 | 5.93 | *Citrus sinensis* |
| Unigene30382_All | 0.01 | 0.54 | 5.75 | *Spinacia oleracea* |
| Unigene31057_All | 0.01 | 0.45 | 5.49 | *Arabidopsis thaliana* |
| CL2829.Contig1_All | 0.06 | 0.25 | 2.06 | *Chenopodium quinoa* |
| CL3030.Contig3_All | 0.71 | 0.01 | -6.15 | *Vitis vinifera* |
| **STAS** | | | | |
| CL6653.Contig2_All | 0.01 | 0.15 | 3.91 | *Stylosanthes hamata* |
| CL576.Contig1_All | 3.20 | 15.25 | 2.25 | *Camellia sinensis* |
| **PHT** | | | | |
| CL5240.Contig3_All | 0.01 | 0.41 | 5.36 | *Medicago truncatula* |
| CL5611.Contig3_All | 0.01 | 0.06 | 2.58 | *Glycine max* |
| Unigene10002_All | 0.71 | 0.01 | -6.15 | *Nicotiana sylvestris* |
| **CLC** | | | | |
| CL3948.Contig2_All | 0.01 | 0.95 | 6.57 | *Vitis vinifera* |
| **BOR** | | | | |
| CL2956.Contig6_All | 3.37 | 1.04 | -1.70 | *Nicotiana sylvestris* |
| **ZnT** | | | | |
| Unigene6915_All | 2.41 | 7.47 | 1.63 | *Vitis vinifera* |
| CL3072.Contig3_All | 0.28 | 1.87 | 2.74 | *Cicer arietinum* |
| Unigene15501_All | 1.82 | 0.01 | -7.51 | *Nelumbo nucifera* |
| CL3501.Contig2_All | 0.36 | 0.01 | -5.17 | *Vitis vinifera* |
| **MOT** | | | | |
| Unigene7283_All | 8.74 | 3.11 | -1.49 | *Nicotiana tomentosiformis* |

**Table S5** DEGs related to ion transport in roots of *A. canescens* under 100 mM NaCl for 6 h. CR6 indicates the leaves of plants treated as a control for 6 h, SR6 indicates the roots of plants treated with 100 mM NaCl for 6 h, and “Fold change” equals log_2_ (FPKM-SR6/FPKM-CR6).

| **Gene ID** | **FPKM-CR6** | **FPKM-SR6** | **Fold change** | **Homologous species** |
| --- | --- | --- | --- | --- |
| **NHX** | | | | |
| Unigene2433_All | 26.04 | 55.39 | 1.09 | *Atriplex halimus* |
| Unigene2571_All | 14.01 | 4.00 | -1.81 | *Mesembryanthemum crystallinum* |
| **SOS1** | | | | |
| CL5512.Contig2_All | 19.48 | 2.84 | -2.78 | *Chenopodium quinoa* |
| **HKT** | | | | |
| CL539.Contig1_All | 21.37 | 2.11 | -3.34 | *Nelumbo nucifera* |
| **KEA** | | | | |
| CL3380.Contig1_All | 0.99 | 60.90 | 5.94 | *Nelumbo nucifera* |
| Unigene14970_All | 13.31 | 4.54 | -1.55 | *Nicotiana tomentosiformis* |
| CL4493.Contig3_All | 3.52 | 0.99 | -1.83 | *Beta vulgaris* |
| **KCO** | | | | |
| Unigene7902_All | 0.01 | 1.60 | 7.32 | *Vitis vinifera* |
| CL6946.Contig2_All | 1.31 | 0.27 | -2.28 | *Vitis vinifera* |
| **SKOR** | | | | |
| CL3066.Contig1_All | 27.69 | 0.27 | -6.68 | *Alternanthera philoxeroides* |
| Unigene39479_All | 0.28 | 0.01 | -4.81 | *Alternanthera philoxeroides* |
| **AKT** | | | | |
| CL5980.Contig1_All | 0.01 | 12.89 | 10.33 | *Solanum tuberosum* |
| CL3322.Contig3_All | 0.01 | 2.36 | 7.88 | *Ricinus communis* |
| CL1419.Contig2_All | 18.88 | 4.64 | -2.02 | *Mesembryanthemum crystallinum* |
| Unigene11168_All | 1.81 | 0.07 | -4.69 | *Glycine max* |
| Unigene1023_All | 18.04 | 0.33 | -5.77 | *Mesembryanthemum crystallinum* |
| Unigene17093_All | 7.10 | 0.01 | -9.47 | *Nicotiana tomentosiformis* |
| CL6985.Contig2_All | 9.42 | 0.01 | -9.88 | *Mesembryanthemum crystallinum* |
| Unigene25880_All | 14.26 | 0.01 | -10.48 | *Mesembryanthemum crystallinum* |
| **KT/HAK/KUP** | | | | |
| CL9787.Contig2_All | 8.61 | 60.56 | 2.81 | *Celosia argentea* |
| Unigene503_All | 1.90 | 13.84 | 2.86 | *Vitis vinifera* |
| Unigene9584_All | 12.89 | 29.87 | 1.21 | *Sesuvium portulacastrum* |
| CL2081.Contig2_All | 3.16 | 0.01 | -8.30 | *Nelumbo nucifera* |
| **NCX** | | | | |
| CL2592.Contig2_All | 13.94 | 57.38 | 2.04 | *Medicago truncatula* |
| CL5484.Contig1_All | 49.74 | 16.24 | -1.61 | *Dianthus caryophyllus* |
| **CNGC** | | | | |
| Unigene12729_All | 0.01 | 6.81 | 9.41 | *Ricinus communis* |
| CL2537.Contig1_All | 18.42 | 3.13 | -2.56 | *Solanum lycopersicum* |
| CL1899.Contig2_All | 4.11 | 0.71 | -2.53 | *Nicotiana tomentosiformis* |
| **CCX** | | | | |
| CL1695.Contig1_All | 0.01 | 8.76 | 9.77 | *Vitis vinifera* |
| CL5759.Contig2_All | 24.18 | 4.95 | -2.29 | *Vitis vinifera* |
| **P-Ca^2+^ ATPase** | | | | |
| Unigene19083_All | 38.18 | 18.21 | -1.07 | *Mesembryanthemum crystallinum* |
| Unigene10043_All | 33.84 | 2.76 | -3.62 | *Sesuvium portulacastrum* |
| CL4614.Contig1_All | 68.50 | 33.87 | -1.02 | *Mesembryanthemum crystallinum* |
| CL1941.Contig1_All | 60.50 | 23.37 | -1.37 | *Chenopodium quinoa* |
| Unigene9206_All | 28.93 | 1.13 | -4.68 | *Vitis vinifera* |
| **P-H^+^ ATPase** | | | | |
| CL2639.Contig4_All | 0.01 | 1.16 | 6.86 | *Eucalyptus grandis* |
| CL6839.Contig1_All | 32.50 | 88.99 | 1.45 | *Sesuvium portulacastrum* |
| CL6971.Contig1_All | 23.06 | 60.06 | 1.38 | *Mesembryanthemum crystallinum* |
| CL8803.Contig2_All | 3.65 | 1.09 | -1.74 | *Sesuvium portulacastrum* |
| Unigene447_All | 14.19 | 6.89 | -1.04 | *Solanum tuberosum* |
| Unigene9556_All | 275.56 | 68.55 | -2.01 | *Sesuvium portulacastrum* |
| Unigene14649_All | 59.27 | 23.87 | -1.31 | *Sesuvium portulacastrum* |
| **V-H^+^ PPase** | | | | |
| CL4556.Contig5_All | 220.07 | 468.10 | 1.09 | *Chenopodium rubrum* |
| CL4556.Contig4_All | 50.64 | 7.04 | -2.85 | *Salicornia europaea* |
| **MGT** | | | | |
| CL1495.Contig7_All | 1.79 | 8.55 | 2.26 | *Cucumis sativus* |
| Unigene4950_All | 7.52 | 15.97 | 1.09 | *Vitis vinifera* |
| CL7136.Contig2_All | 28.49 | 5.04 | -2.50 | *Suaeda glauca* |
| CL3103.Contig3_All | 6.58 | 2.88 | -1.19 | *Chenopodium quinoa* |
| Unigene29722_All | 0.41 | 0.01 | -5.36 | *Nelumbo nucifera* |
| Unigene11959_All | 3.67 | 0.46 | -3.00 | *Vitis vinifera* |
| CL9565.Contig2_All | 5.36 | 1.80 | -1.57 | *Cucumis sativus* |
| **AMT** | | | | |
| Unigene10940_All | 0.01 | 4.34 | 8.76 | *Medicago truncatula* |
| Unigene1346_All | 0.07 | 9.82 | 7.13 | *Alternanthera philoxeroides* |
| Unigene10919_All | 0.62 | 3.89 | 2.65 | *Alternanthera philoxeroides* |
| Unigene1739_All | 8.86 | 0.34 | -4.70 | *Eucalyptus grandis* |
| **NRT** | | | | |
| CL6490.Contig1_All | 0.83 | 35.04 | 5.40 | *Vitis vinifera* |
| CL3030.Contig6_All | 0.55 | 4.59 | 3.06 | *Vitis vinifera* |
| CL3698.Contig3_All | 0.91 | 5.36 | 2.56 | *Spinacia oleracea* |
| CL6484.Contig1_All | 23.19 | 2.00 | -3.54 | *Vitis vinifera* |
| CL2228.Contig2_All | 10.75 | 0.91 | -3.56 | *Theobroma cacao* |
| CL2829.Contig1_All | 6.65 | 0.13 | -5.68 | *Chenopodium quinoa* |
| Unigene31327_All | 1.79 | 0.01 | -7.48 | *Solanum lycopersicum* |
| CL8745.Contig1_All | 26.86 | 0.06 | -8.81 | *Ricinus communis* |
| Unigene12841_All | 9.87 | 0.01 | -9.95 | *Citrus sinensis* |
| **STAS** | | | | |
| CL576.Contig7_All | 10.80 | 55.49 | 2.36 | *Camellia sinensis* |
| CL4513.Contig1_All | 1.17 | 4.38 | 1.90 | *Sesuvium portulacastrum* |
| CL6653.Contig2_All | 33.59 | 0.15 | -7.81 | *Stylosanthes hamata* |
| **PHT** | | | | |
| CL7475.Contig1_All | 0.29 | 13.96 | 5.59 | *Spinacia oleracea* |
| CL5240.Contig1_All | 29.29 | 0.01 | -11.52 | *Medicago sativa* |
| CL5611.Contig1_All | 6.88 | 0.01 | -9.43 | *Citrus sinensis* |
| Unigene5615_All | 5.00 | 0.01 | -8.97 | *Medicago truncatula* |
| Unigene10002_All | 2.92 | 0.30 | -3.28 | *Nicotiana sylvestris* |
| Unigene4292_All | 24.70 | 4.11 | -2.59 | *Vitis vinifera* |
| **CLC** | | | | |
| CL3948.Contig2_All | 0.37 | 3.23 | 3.13 | *Vitis vinifera* |
| CL2875.Contig3_All | 22.34 | 7.06 | -1.66 | *Vitis vinifera* |
| CL1933.Contig2_All | 12.53 | 3.71 | -1.76 | *Eucalyptus grandis* |
| **SLAH** | | | | |
| Unigene12189_All | 47.42 | 0.07 | -9.40 | *Morus notabilis* |
| **CTR** | | | | |
| Unigene14371_All | 0.01 | 8.26 | 9.69 | *Theobroma cacao* |
| Unigene1692_All | 18.81 | 0.13 | -7.18 | *Theobroma cacao* |
| Unigene464_All | 9.77 | 0.45 | -4.44 | *Ricinus communis* |
| CL6889.Contig2_All | 572.77 | 112.28 | -2.35 | *Chenopodium quinoa* |
| **BOR** | | | | |
| CL2956.Contig3_All | 17.45 | 2.56 | -2.77 | *Theobroma cacao* |
| Unigene4180_All | 28.61 | 5.29 | -2.44 | *Cucumis sativus* |
| CL6313.Contig1_All | 43.01 | 9.29 | -2.21 | *Vitis vinifera* |
| Unigene32119_All | 1.86 | 0.01 | -7.54 | *Cucumis melo* |
| **ZnT** | | | | |
| Unigene26807_All | 0.01 | 8.97 | 9.81 | *Nicotiana tabacum* |
| Unigene6915_All | 7.73 | 20.64 | 1.42 | *Vitis vinifera* |
| CL3072.Contig3_All | 78.08 | 0.01 | -12.93 | *Cicer arietinum* |
| CL3501.Contig1_All | 7.46 | 1.40 | -2.41 | *Vitis vinifera* |
| Unigene6856_All | 22.60 | 4.53 | -2.32 | *Solanum lycopersicum* |
| Unigene13213_All | 46.84 | 21.15 | -1.15 | *Chenopodium quinoa* |
| CL9112.Contig1_All | 10.88 | 5.04 | -1.11 | *Medicago truncatula* |
| Unigene34495_All | 7.71 | 1.56 | -2.31 | *Theobroma cacao* |
| **MOT** | | | | |
| Unigene7283_All | 27.80 | 1.40 | -4.31 | *Nicotiana tomentosiformis* |
| CL2778.Contig2_All | 35.87 | 7.45 | -2.27 | *Nicotiana tomentosiformis* |

**Table S6** DEGs related to ion transport in roots of *A. canescens* under 100 mM NaCl for 24 h. CR24 indicates the roots of plants treated as a control for 24 h, SR24 indicates the roots of plants treated with 100 mM NaCl for 24 h, and “Fold change” equals log_2_ (FPKM-SR24/FPKM-CR24).

| **Gene ID** | **FPKM-CR24** | **FPKM-SR24** | **Fold change** | **Homologous species** |
| --- | --- | --- | --- | --- |
| **SOS1** | | | | |
| CL5512.Contig2_All | 5.33 | 12.24 | 1.20 | *Chenopodium quinoa* |
| **HKT** | | | | |
| CL539.Contig1_All | 29.60 | 5.15 | -2.52 | *Nelumbo nucifera* |
| Unigene8387_All | 0.67 | 0.01 | -6.07 | *Mesembryanthemum crystallinum* |
| **KCO** | | | | |
| CL6946.Contig2_All | 0.77 | 3.00 | 1.96 | *Vitis vinifera* |
| **SKOR** | | | | |
| Unigene39479_All | 0.01 | 1.55 | 7.28 | *Alternanthera philoxeroides* |
| **AKT** | | | | |
| CL3322.Contig3_All | 0.01 | 3.10 | 8.28 | *Ricinus communis* |
| CL6985.Contig2_All | 0.01 | 1.65 | 5.37 | *Mesembryanthemum crystallinum* |
| Unigene23969_All | 0.01 | 0.28 | 4.81 | *Mesembryanthemum crystallinum* |
| Unigene23528_All | 0.61 | 0.01 | -5.93 | *Mesembryanthemum crystallinum* |
| **KT/HAK/KUP** | | | | |
| CL2081.Contig2_All | 0.01 | 1.93 | 7.59 | *Nelumbo nucifera* |
| **CNGC** | | | | |
| Unigene19313_All | 0.01 | 0.40 | 5.32 | *Vitis vinifera* |
| Unigene12729_All | 0.01 | 0.15 | 3.91 | *Ricinus communis* |
| Unigene142_All | 0.08 | 0.49 | 2.61 | *Cicer arietinum* |
| CL1899.Contig4_All | 0.44 | 2.06 | 2.23 | *Nicotiana tomentosiformis* |
| CL4135.Contig7_All | 0.29 | 0.01 | -4.86 | *Vitis vinifera* |
| **CCX** | | | | |
| CL1695.Contig1_All | 0.01 | 4.68 | 8.87 | *Vitis vinifera* |
| CL5759.Contig1_All | 2.71 | 0.01 | -8.08 | *Eucalyptus grandis* |
| **P-H^+^ ATPase** | | | | |
| CL8125.Contig1_All | 0.01 | 1.14 | 6.83 | *Populus trichocarpa* |
| CL2639.Contig9_All | 0.01 | 0.87 | 6.44 | *Eucalyptus grandis* |
| Unigene33192_All | 0.62 | 1.91 | 1.62 | *Glycine max* |
| Unigene14516_All | 0.50 | 1.11 | 1.15 | *Populus trichocarpa* |
| **V-H^+^ PPase** | | | | |
| CL7188.Contig1_All | 0.01 | 1.77 | 7.47 | *Vitis vinifera* |
| **MGT** | | | | |
| CL7136.Contig3_All | 0.99 | 3.38 | 1.77 | *Suaeda glauca* |
| Unigene11959_All | 1.16 | 2.96 | 1.35 | *Vitis vinifera* |
| CL1495.Contig7_All | 4.40 | 0.01 | -8.78 | *Cucumis sativus* |
| **AMT** | | | | |
| Unigene10940_All | 0.01 | 0.08 | 3.00 | *Medicago truncatula* |
| Unigene10919_All | 0.09 | 0.46 | 2.35 | *Alternanthera philoxeroides* |
| Unigene1346_All | 0.13 | 0.47 | 1.85 | *Alternanthera philoxeroides* |
| Unigene9694_All | 0.74 | 1.53 | 1.05 | *Alternanthera philoxeroides* |
| **NRT** | | | | |
| CL8745.Contig2_All | 0.01 | 2.96 | 8.21 | *Ricinus communis* |
| Unigene37170_All | 0.01 | 0.68 | 6.09 | *Cicer arietinum* |
| Unigene27723_All | 0.53 | 3.37 | 2.67 | *Ricinus communis* |
| Unigene32523_All | 0.29 | 1.81 | 2.64 | *Solanum lycopersicum* |
| Unigene27369_All | 0.30 | 1.55 | 2.37 | *Spinacia oleracea* |
| Unigene31327_All | 0.72 | 2.78 | 1.95 | *Solanum lycopersicum* |
| Unigene8885_All | 0.37 | 1.15 | 1.64 | *Morus notabilis* |
| CL3698.Contig3_All | 1.42 | 4.11 | 1.53 | *Spinacia oleracea* |
| Unigene26957_All | 0.64 | 1.32 | 1.04 | *Spinacia oleracea* |
| Unigene30382_All | 1.70 | 3.50 | 1.04 | *Spinacia oleracea* |
| Unigene31057_All | 1.41 | 0.48 | -1.55 | *Arabidopsis thaliana* |
| Unigene36682_All | 0.46 | 0.01 | -5.52 | *Spinacia oleracea* |
| Unigene6575_All | 1.54 | 0.01 | -7.27 | *Gossypium hirsutum* |
| Unigene4441_All | 3.35 | 0.01 | -8.39 | *Solanum tuberosum* |
| Unigene26958_All | 3.92 | 0.01 | -8.61 | *Spinacia oleracea* |
| **STAS** | | | | |
| CL4513.Contig4_All | 3.70 | 8.46 | 1.19 | *Sesuvium portulacastrum* |
| CL576.Contig3_All | 0.39 | 0.81 | 1.05 | *Camellia sinensis* |
| **PHT** | | | | |
| CL5240.Contig2_All | 0.01 | 1.23 | 6.94 | *Medicago truncatula* |
| Unigene10002_All | 0.99 | 3.58 | 1.85 | *Nicotiana sylvestris* |
| **BOR** | | | | |
| CL2956.Contig6_All | 29.40 | 13.13 | -1.16 | *Nicotiana sylvestris* |
| **ZnT** | | | | |
| Unigene26807_All | 1.68 | 7.29 | 2.12 | *Nicotiana tabacum* |
| Unigene15501_All | 1.09 | 2.24 | 1.04 | *Nelumbo nucifera* |

**Table S7** DEGs related to organic osmolytes synthesis in leaves of *A. canescens* under 100 mM NaCl for 6 h. CL6 indicates the leaves of plants treated as control for 6 h, SL6 indicates the leaves of plants treated with 100 mM NaCl for 6 h, and “Fold change” equals log_2_ (FPKM-SL6/FPKM-CL6).

| **Gene ID** | **FPKM-CL6** | **FPKM-SL6** | **Fold change** | **Homologous species** |
| --- | --- | --- | --- | --- |
| **P5CS** |  |  |  |  |
| Unigene27678_All | 0.01 | 3.19 | 8.32 | *Vitis vinifera* |
| **OAT** | | | | |
| Unigene3360_All | 5.77 | 14.00 | 1.28 | *Malus domestica* |
| **GDH** | | | | |
| CL1754.Contig1_All | 14.58 | 144.14 | 3.31 | *Ricinus communis* |
| Unigene27948_All | 22.84 | 213.72 | 3.23 | *Glycine max* |
| **GOGAT** | | | | |
| CL9819.Contig4_All | 0.01 | 9.38 | 9.87 | *Vitis vinifera* |
| Unigene31175_All | 0.77 | 38.20 | 5.63 | *Populus trichocarpa* |
| Unigene955_All | 166.74 | 26.81 | -2.64 | *Spinacia oleracea* |
| **BADH** | | | | |
| CL8998.Contig2_All | 40.31 | 85.19 | 1.08 | *Amaranthus hypochondriacus* |
| **CMO** | | | | |
| CL1302.Contig2_All | 2.66 | 6.01 | 1.18 | *Oryza sativa* |
| **PEAMT** | | | | |
| Unigene8968_All | 211.76 | 22.68 | -3.22 | *Atriplex canescens* |
| CL5639.Contig1_All | 277.66 | 13.28 | -4.39 | *Atriplex canescens* |
| Unigene41622_All | 62.24 | 14.86 | -2.07 | *Spinacia oleracea* |
| **INV** | | | | |
| CL879.Contig4_All | 0.01 | 19.93 | 10.96 | *Chenopodium rubrum* |
| CL7150.Contig1_All | 0.01 | 7.57 | 9.56 | *Beta vulgaris* |
| CL879.Contig1_All | 0.01 | 6.22 | 9.28 | *Chenopodium rubrum* |
| Unigene28636_All | 0.01 | 4.37 | 8.77 | *Beta vulgaris* |
| Unigene36824_All | 0.01 | 0.53 | 5.73 | *Beta vulgaris* |
| Unigene14974_All | 4.70 | 39.75 | 3.08 | *Nelumbo nucifera* |
| CL910.Contig2_All | 1.39 | 7.18 | 2.37 | *Camellia sinensis* |
| Unigene774_All | 19.96 | 48.29 | 1.27 | *Nelumbo nucifera* |
| Unigene27182_All | 1.23 | 2.50 | 1.02 | *Beta vulgaris* |
| CL4395.Contig1_All | 1.42 | 0.15 | -3.24 | *Beta vulgaris* |
| CL85.Contig5_All | 2.26 | 0.01 | -7.82 | *Beta vulgaris* |
| **SuSy** | | | | |
| CL1644.Contig3_All | 0.01 | 2.72 | 8.09 | *Nelumbo nucifera* |
| CL2395.Contig2_All | 25.13 | 418.07 | 4.06 | *Chenopodium rubrum* |
| CL796.Contig1_All | 2.94 | 6.65 | 1.18 | *Beta vulgaris* |
| **SPS** | | | | |
| CL9220.Contig3_All | 0.01 | 0.65 | 6.02 | *Spinacia oleracea* |
| Unigene18731_All | 8.90 | 3.82 | -1.22 | *Beta vulgaris* |
| **TPS** | | | | |
| Unigene31484_All | 0.01 | 0.66 | 6.04 | *Ricinus communis* |
| CL6053.Contig2_All | 0.53 | 7.66 | 3.85 | *Vitis vinifera* |
| CL6691.Contig2_All | 0.49 | 2.45 | 2.32 | *Nelumbo nucifera* |
| Unigene13729_All | 29.29 | 59.44 | 1.02 | *Nelumbo nucifera* |
| CL9718.Contig1_All | 36.32 | 280.85 | 2.95 | *Vitis vinifera* |
| Unigene1043_All | 0.19 | 0.38 | 1.00 | *Solanum lycopersicum* |
| CL6902.Contig2_All | 1.94 | 0.01 | -7.60 | *Camellia sinensis* |
| **AMS** | | | | |
| CL2655.Contig3_All | 0.01 | 3.54 | 8.47 | *Vitis vinifera* |
| CL6110.Contig1_All | 0.01 | 2.84 | 8.15 | *Theobroma cacao* |
| Unigene26924_All | 0.01 | 0.27 | 4.75 | *Vitis vinifera* |
| CL1868.Contig3_All | 1.52 | 5.88 | 1.95 | *Vitis vinifera* |
| CL1130.Contig1_All | 8.26 | 1.40 | -2.56 | *Nelumbo nucifera* |
| CL1235.Contig1_All | 6.08 | 2.56 | -1.25 | *Eucalyptus grandis* |
| CL261.Contig9_All | 5.99 | 2.72 | -1.14 | *Theobroma cacao* |
| CL4038.Contig1_All | 119.74 | 33.25 | -1.85 | *Nelumbo nucifera* |
| CL6283.Contig1_All | 422.43 | 55.97 | -2.92 | *Actinidia chinensis* |
| CL988.Contig2_All | 5.23 | 0.95 | -2.46 | *Morus notabilis* |
| Unigene18636_All | 0.68 | 0.01 | -6.09 | *Camellia sinensis* |
| Unigene18937_All | 17.93 | 3.03 | -2.56 | *Citrus sinensis* |
| Unigene7549_All | 8.06 | 0.63 | -3.68 | *Glycine max* |
| **SS** | | | | |
| CL3413.Contig1_All | 0.01 | 7.97 | 9.64 | *Amaranthus cruentus* |
| Unigene30135_All | 0.01 | 2.25 | 7.81 | *Amaranthus cruentus* |
| CL2996.Contig1_All | 92.44 | 7.98 | -3.53 | *Nelumbo nucifera* |
| Unigene19053_All | 8.37 | 0.34 | -4.62 | *Chenopodium quinoa* |
| CL549.Contig1_All | 72.58 | 27.14 | -1.42 | *Brassica rapa* |
| Unigene16272_All | 44.10 | 8.01 | -2.46 | *Amaranthus cruentus* |
| CL327.Contig2_All | 12.99 | 2.87 | -2.18 | *Vitis vinifera* |
| CL1393.Contig1_All | 2.82 | 0.33 | -3.10 | *Vitis vinifera* |
| **MD** | | | | |
| CL4146.Contig3_All | 0.73 | 173.38 | 7.89 | *Mesembryanthemum crystallinum* |
| CL5740.Contig4_All | 1.28 | 30.35 | 4.57 | *Mesembryanthemum crystallinum* |

**Table S8** DEGs related to organic osmolytes synthesis in leaves of *A. canescens* under 100 mM NaCl for 24 h. CL24 indicates the leaves of plants treated as control for 24 h, SL24 indicates the leaves of plants treated with 100 mM NaCl for 24 h, and “Fold change” equals log_2_ (FPKM-SL24/FPKM-CL24).

| **Gene ID** | **FPKM-CL24** | **FPKM-SL24** | **Fold change** | **Homologous species** |
| --- | --- | --- | --- | --- |
| **P5CS** |  |  |  |  |
| Unigene27678_All | 0.01 | 0.34 | 5.09 | *Vitis vinifera* |
| **OAT** | | | | |
| CL1780.Contig4_All | 6.74 | 24.04 | 1.83 | *Solanum lycopersicum* |
| **GDH** | | | | |
| Unigene27948_All | 28.27 | 13.37 | -1.08 | *Glycine max* |
| **GOGAT** | | | | |
| CL9819.Contig4_All | 0.01 | 0.40 | 5.32 | *Vitis vinifera* |
| **BADH** | | | | |
| CL8998.Contig2_All | 0.03 | 3.31 | 6.79 | *Atriplex centralasiatica* |
| **CMO** | | | | |
| CL1302.Contig2_All | 0.19 | 2.11 | 3.47 | *Pyrus betulifolia* |
| **INV** | | | | |
| CL85.Contig5_All | 0.01 | 1.71 | 7.42 | *Beta vulgaris* |
| CL879.Contig1_All | 0.01 | 0.08 | 3.00 | *Beta vulgaris* |
| CL4395.Contig1_All | 0.58 | 2.00 | 1.79 | *Beta vulgaris* |
| Unigene27182_All | 1.72 | 0.80 | -1.10 | *Beta vulgaris* |
| CL6919.Contig5_All | 4.66 | 1.91 | -1.29 | *Theobroma cacao* |
| CL7150.Contig1_All | 0.45 | 0.01 | -5.49 | *Beta vulgaris* |
| **SuSy** | | | | |
| CL1644.Contig3_All | 0.01 | 1.74 | 7.44 | *Nelumbo nucifera* |
| CL796.Contig1_All | 6.08 | 17.62 | 1.54 | *Beta vulgaris* |
| Unigene40753_All | 1.55 | 0.20 | -2.95 | *Hevea brasiliensis* |
| CL4061.Contig1_All | 2.25 | 0.74 | -1.60 | *Arabidopsis thaliana* |
| **SPS** | | | | |
| CL9220.Contig3_All | 3.71 | 12.06 | 1.70 | *Spinacia oleracea* |
| **TPS** | | | | |
| Unigene1043_All | 0.01 | 0.71 | 6.15 | *Solanum lycopersicum* |
| CL6902.Contig2_All | 1.81 | 6.63 | 1.87 | *Camellia sinensis* |
| **AMS** | | | | |
| CL2655.Contig3_All | 0.01 | 0.76 | 6.25 | *Vitis vinifera* |
| CL6110.Contig1_All | 0.01 | 0.73 | 6.19 | *Theobroma cacao* |
| CL261.Contig1_All | 0.01 | 0.32 | 5.00 | *Theobroma cacao* |
| Unigene7295_All | 23.68 | 70.13 | 1.57 | *Nicotiana tomentosiformis* |
| Unigene18636_All | 0.64 | 1.29 | 1.01 | *Camellia sinensis* |
| CL1868.Contig2_All | 1.02 | 0.01 | -6.67 | *Nicotiana tomentosiformis* |
| **SS** | | | | |
| Unigene27067_All | 0.49 | 4.56 | 3.22 | *Lotus japonicus* |
| Unigene19053_All | 2.22 | 10.00 | 2.17 | *Chenopodium quinoa* |
| CL1393.Contig4_All | 5.95 | 1.06 | -2.49 | *Vitis vinifera* |
| **MD** | | | | |
| CL4146.Contig3_All | 0.19 | 0.78 | 2.04 | *Mesembryanthemum crystallinum* |
| CL5740.Contig4_All | 4.93 | 1.90 | -1.38 | *Mesembryanthemum crystallinum* |

**Table S9** DEGs related to photosynthesis in leaves of *A. canescens* under 100 mM NaCl for 6 h. CL6 indicates the leaves of plants treated as control for 6 h, SL6 indicates the leaves of plants treated with 100 mM NaCl for 6 h, “Fold change” equals log_2_ (FPKM-SL6/FPKM-CL6).

| **Gene ID** | **FPKM-CL6** | **FPKM-SL6** | **Fold change** | **Homologous species** |
| --- | --- | --- | --- | --- |
| **Photosystem II** | | | | |
| Unigene31059_All | 0.01 | 1.05 | 6.71 | *Oryza sativa* |
| Unigene7309_All | 44.72 | 9.01 | -2.31 | *Citrus sinensis* |
| CL272.Contig1_All | 18.04 | 2.89 | -2.64 | *Citrus sinensis* |
| Unigene14383_All | 17.21 | 2.71 | -2.67 | *Arabidopsis thaliana* |
| CL2334.Contig1_All | 43.62 | 5.36 | -3.02 | *Pelargonium cotyledonis* |
| CL6901.Contig1_All | 128.97 | 14.04 | -3.20 | *Solanum lycopersicum* |
| Unigene1699_All | 11.18 | 0.86 | -3.70 | *Citrus sinensis* |
| CL3934.Contig2_All | 16.58 | 0.66 | -4.65 | *Vitis vinifera* |
| Unigene2053_All | 2.35 | 0.07 | -5.07 | *Beta vulgaris* |
| Unigene10371_All | 83.29 | 1.95 | -5.42 | *Nicotiana sylvestris* |
| CL5305.Contig1_All | 1068.95 | 17.79 | -5.91 | *Spinacia oleracea* |
| CL6311.Contig1_All | 7.56 | 0.06 | -6.98 | *Ricinus communis* |
| Unigene8762_All | 53.96 | 0.33 | -7.35 | *Erythranthe guttata* |
| CL4004.Contig1_All | 2188.21 | 12.28 | -7.48 | *Vitis vinifera* |
| Unigene4996_All | 2501.96 | 11.10 | -7.82 | *Nicotiana sylvestris* |
| CL4550.Contig3_All | 2356.56 | 6.76 | -8.16 | *Nicotiana sylvestris* |
| CL415.Contig7_All | 2522.73 | 7.39 | -8.42 | *Spinacia oleracea* |
| Unigene3638_All | 2802.37 | 8.06 | -8.44 | *Spinacia oleracea* |
| CL329.Contig1_All | 4.36 | 0.01 | -8.77 | *Chenopodium quinoa* |
| Unigene3330_All | 3168.95 | 6.47 | -8.94 | *Salicornia europaea* |
| CL186.Contig2_All | 1873.22 | 3.39 | -9.11 | *Spinacia oleracea* |
| CL4412.Contig1_All | 2612.98 | 4.14 | -9.30 | *Spinacia oleracea* |
| CL5242.Contig1_All | 2183.02 | 2.42 | -9.82 | *Salicornia europaea* |
| Unigene13245_All | 1035.3 | 1.13 | -9.84 | *Solanum lycopersicum* |
| Unigene14068_All | 610.77 | 0.51 | -10.23 | *Chenopodium quinoa* |
| Unigene17728_All | 617.28 | 0.35 | -10.78 | *Spinacia oleracea* |
| CL3624.Contig1_All | 629.39 | 0.31 | -10.99 | *Morus notabilis* |
| Unigene16050_All | 701.30 | 0.33 | -11.05 | *Vitis vinifera* |
| Unigene17535_All | 324.59 | 0.01 | -14.99 | *Citrus sinensis* |
| Unigene12234_All | 441.23 | 0.01 | -15.43 | *Spinacia oleracea* |
| **Cytochrome b_6/_f complex** | | | | |
| Unigene40452_All | 0.01 | 0.66 | 6.04 | *Vaccinium macrocarpon* |
| Unigene22934_All | 0.43 | 0.01 | -5.43 | *Medicago truncatula* |
| Unigene4558_All | 2.61 | 0.52 | -2.33 | *Beta vulgaris* |
| **Photosystem I** | | | | |
| Unigene14296_All | 3.90 | 13.54 | 1.80 | *Vitis vinifera* |
| Unigene22084_All | 0.95 | 0.32 | -1.57 | *Portulaca oleracea* |
| Unigene18662_All | 3.77 | 1.13 | -1.74 | *Chenopodium quinoa* |
| Unigene18516_All | 1.21 | 0.21 | -2.53 | *Beta vulgaris* |
| Unigene10241_All | 3.00 | 0.46 | -2.71 | *Portulacaria afra* |
| CL759.Contig1_All | 2.43 | 0.34 | -2.84 | *Morus notabilis* |
| CL9242.Contig1_All | 9.08 | 0.37 | -4.62 | *Medicago truncatula* |
| CL4525.Contig1_All | 0.60 | 0.01 | -5.91 | *Portulaca oleracea* |
| CL8047.Contig1_All | 0.62 | 0.01 | -5.95 | *Portulaca oleracea* |
| Unigene1257_All | 1933 | 30.72 | -5.98 | *Spinacia oleracea* |
| Unigene20247_All | 0.66 | 0.01 | -6.04 | *Lindenbergia philippensis* |
| CL4449.Contig2_All | 2356.39 | 33.61 | -6.13 | *Spinacia oleracea* |
| Unigene22784_All | 1.35 | 0.01 | -7.08 | *Carpobrotus chilensis* |
| Unigene7008_All | 2.43 | 0.01 | -7.92 | *Vitis vinifera* |
| Unigene12274_All | 1518.51 | 5.27 | -8.17 | *Spinacia oleracea* |
| CL236.Contig1_All | 815.89 | 2.70 | -8.24 | *Spinacia oleracea* |
| CL9062.Contig2_All | 3.17 | 0.01 | -8.31 | *Portulaca oleracea* |
| Unigene3636_All | 2086.82 | 1.49 | -10.45 | *Spinacia oleracea* |
| Unigene6698_All | 1645.82 | 1.17 | -10.46 | *Spinacia oleracea* |
| Unigene15753_All | 368.22 | 0.12 | -11.58 | *Morus notabilis* |
| Unigene5238_All | 2085.23 | 0.15 | -13.76 | *Spinacia oleracea* |
| **Ferredoxin** | | | | |
| Unigene5352_All | 7.29 | 173.25 | 4.57 | *Ricinus communis* |
| Unigene6812_All | 101.68 | 384.42 | 1.92 | *Vitis vinifera* |
| Unigene3533_All | 130.41 | 16.13 | -3.02 | *Spinacia oleracea* |
| Unigene15767_All | 76.02 | 6.08 | -3.64 | *Ricinus communis* |
| CL90.Contig1_All | 48.87 | 0.85 | -5.85 | *Mesembryanthemum crystallinum* |
| CL7286.Contig1_All | 727.86 | 4.31 | -7.40 | *Spinacia oleracea* |
| Unigene5233_All | 50.96 | 0.17 | -8.23 | *Theobroma cacao* |
| Unigene12466_All | 2112.85 | 0.83 | -11.31 | *Mesembryanthemum crystallinum* |
| **Thylakoid membrane ATP synthase** | | | | |
| CL1029.Contig1_All | 0.56 | 2.10 | 1.91 | *Medicago truncatula* |
| CL1018.Contig2_All | 8.67 | 0.26 | -5.06 | *Beta vulgaris* |
| Unigene13973_All | 384.93 | 5.21 | -6.21 | *Spinacia oleracea* |
| Unigene17493_All | 461.72 | 2.50 | -7.53 | *Spinacia oleracea* |
| Unigene14007_All | 894.63 | 0.66 | -10.40 | *Spinacia oleracea* |
| **Carbon fixation** | | | | |
| Unigene12193_All | 0.96 | 293.11 | 8.25 | *Populus trichocarpa* |
| CL2649.Contig3_All | 39.39 | 173.04 | 2.14 | *Suaeda glauca* |
| Unigene34895_All | 0.01 | 0.35 | 5.13 | *Rhododendron micranthum* |
| Unigene1403_All | 5.36 | 86.31 | 4.01 | *Vitis vinifera* |
| Unigene14361_All | 4.32 | 45.28 | 3.39 | *Nelumbo nucifera* |
| Unigene6890_All | 3.52 | 35.90 | 3.35 | *Vitis vinifera* |
| Unigene19952_All | 9.88 | 78.23 | 2.99 | *Vitis vinifera* |
| CL6958.Contig4_All | 18.74 | 100.17 | 3.84 | *Ricinus communis* |
| CL791.Contig2_All | 3.95 | 15.11 | 1.94 | *Nelumbo nucifera* |
| CL7555.Contig1_All | 12.67 | 37.60 | 1.57 | *Amaranthus hypochondriacus* |
| CL6760.Contig2_All | 2.67 | 7.26 | 1.44 | *Ricinus communis* |
| CL6022.Contig2_All | 12.71 | 33.95 | 1.42 | *Vitis vinifera* |
| CL8006.Contig1_All | 8.89 | 19.57 | 1.14 | *Vitis vinifera* |
| CL7761.Contig1_All | 29.62 | 64.50 | 1.12 | *Nelumbo nucifera* |
| CL3863.Contig2_All | 358.06 | 170.22 | -1.07 | *Oryza sativa* |
| Unigene3773_All | 5.32 | 2.18 | -1.29 | *Vitis vinifera* |
| Unigene9780_All | 450.75 | 166.28 | -1.44 | *Theobroma cacao* |
| Unigene1772_All | 28.99 | 10.69 | -1.44 | *Theobroma cacao* |
| Unigene7172_All | 8.65 | 2.43 | -1.83 | *Capsicum annuum* |
| CL4895.Contig1_All | 783.40 | 191.66 | -2.03 | *Medicago truncatula* |
| Unigene1593_All | 51.74 | 12.27 | -2.08 | *Spinacia oleracea* |
| CL4619.Contig1_All | 3.46 | 0.78 | -2.15 | *Eucalyptus grandis* |
| Unigene12101_All | 242.84 | 54.41 | -2.16 | *Eucalyptus grandis* |
| Unigene13979_All | 270.61 | 58.42 | -2.21 | *Spinacia oleracea* |
| CL6148.Contig1_All | 3.67 | 0.69 | -2.41 | *Vitis vinifera* |
| CL5319.Contig2_All | 240.36 | 41.83 | -2.52 | *Spinacia oleracea* |
| CL519.Contig3_All | 102.56 | 10.98 | -3.22 | *Theobroma cacao* |
| CL9100.Contig1_All | 107.33 | 9.74 | -3.46 | *Spinacia oleracea* |
| Unigene18015_All | 197.21 | 13.12 | -3.91 | *Citrus sinensis* |
| Unigene8864_All | 397.13 | 14.96 | -4.73 | *Spinacia oleracea* |
| Unigene861_All | 0.29 | 0.01 | -4.86 | *Flaveria trinervia* |
| Unigene3148_All | 35.69 | 1.22 | -4.87 | *Vitis vinifera* |
| CL1287.Contig1_All | 75.89 | 1.97 | -5.27 | *Spinacia oleracea* |
| Unigene10764_All | 58.04 | 1.01 | -5.84 | [*Solanum lycopersicum*](https://blast.ncbi.nlm.nih.gov/Blast.cgi#alnHdr_1090863992) |
| CL3000.Contig1_All | 1314.28 | 6.65 | -7.63 | *Chenopodium album* |
| CL2649.Contig2_All | 1342.40 | 3.02 | -8.80 | *Alternanthera sessilis* |
| CL3561.Contig2_All | 3428.81 | 5.76 | -9.22 | *Amaranthus hypochondriacus* |
| CL8974.Contig1_All | 2049.95 | 2.39 | -9.74 | *Spinacia oleracea* |
| Unigene10707_All | 6011.67 | 6.07 | -9.95 | *Eucalyptus grandis* |
| CL5649.Contig2_All | 1213.10 | 0.42 | -11.50 | *Spinacia oleracea* |
| CL5885.Contig1_All | 1111.05 | 0.29 | -11.90 | *Spinacia oleracea* |
| CL7642.Contig1_All | 45.25 | 0.01 | -12.14 | *Salicornia herbacea* |
| Unigene29041_All | 1477.10 | 0.14 | -13.37 | *Spinacia oleracea* |
| **Chlorophyll biosynthesis** | | | | |
| CL3679.Contig1_All | 9.63 | 4.34 | -1.15 | *Citrus sinensis* |
| Unigene11075_All | 12.12 | 4.44 | -1.45 | *Vitis vinifera* |
| Unigene1355_All | 61.80 | 17.37 | -1.83 | *Gossypium hirsutum* |
| Unigene17852_All | 67.33 | 18.59 | -1.86 | *Spinacia oleracea* |
| Unigene14212_All | 43.59 | 11.66 | -1.90 | *Spinacia oleracea* |
| Unigene13195_All | 76.51 | 13.22 | -2.53 | *Nicotiana tomentosiformis* |
| Unigene17713_All | 57.23 | 8.50 | -2.75 | *Ricinus communis* |
| Unigene15828_All | 84.27 | 11.27 | -2.90 | *Nicotiana tomentosiformis* |
| Unigene13920_All | 171.16 | 7.80 | -4.46 | *Solanum tuberosum* |
| Unigene6885_All | 129.51 | 1.85 | -6.13 | *Nicotiana sylvestris* |
| Unigene17707_All | 769.64 | 7.89 | -6.61 | *Populus trichocarpa* |
| CL8468.Contig1_All | 2.86 | 0.01 | -8.16 | *Spinacia oleracea* |
| Unigene1034_All | 337.18 | 0.50 | -9.40 | *Camellia sinensis* |
| **Chlorophyll catabolism** | | | | |
| CL2622.Contig1_All | 52.82 | 13.51 | -1.97 | *Theobroma cacao* |
| Unigene14870_All | 1.42 | 0.24 | -2.56 | *Chenopodium album* |
| CL2749.Contig1_All | 6.31 | 1.01 | -2.64 | *Medicago truncatula* |
| Unigene16044_All | 40.24 | 2.48 | -4.02 | *Eucalyptus grandis* |
| Unigene6738_All | 30.41 | 0.18 | -7.40 | *Citrus sinensis* |

**Table S10** DEGs related to photosynthesis in leaves of *A. canescens* under 100 mM NaCl for 24 h. CL24 indicates the leaves of plants treated as control for 24 h, SL24 indicates the leaves of plants treated with 100 mM NaCl for 24 h, and “Fold change” equals log_2_ (FPKM-SL24/FPKM-CL24).

| **Gene ID** | **FPKM-CL24** | **FPKM-SL24** | **Fold change** | **Homologous species** |
| --- | --- | --- | --- | --- |
| **Photosystem II** | | | | |
| CL4550.Contig3_All | 4.19 | 8.67 | 1.05 | *Malus domestica* |
| Unigene173_All | 1.08 | 2.21 | 1.03 | *Medicago truncatula* |
| CL6293.Contig3_All | 3.54 | 1.53 | -1.21 | *Spinacia oleracea* |
| CL329.Contig3_All | 3.96 | 1.28 | -1.63 | *Chenopodium quinoa* |
| CL272.Contig2_All | 1.36 | 0.01 | -7.09 | *Citrus sinensis* |
| CL4004.Contig3_All | 10.81 | 0.01 | -10.08 | *Vitis vinifera* |
| **Cytochrome b_6/_f complex** | | | | |
| Unigene7017_All | 0.46 | 1.38 | 1.58 | *Eucalyptus curtisii* |
| Unigene40452_All | 0.62 | 0.01 | -5.95 | *Vaccinium macrocarpon* |
| **Photosystem I** | | | | |
| Unigene25839_All | 0.01 | 1.44 | 7.17 | *Suaeda salsa* |
| Unigene18662_All | 1.78 | 5.72 | 1.68 | *Morus mongolica* |
| Unigene10241_All | 1.09 | 2.85 | 1.39 | *Portulacaria afra* |
| Unigene25231_All | 0.62 | 1.24 | 1.00 | *Chenopodium album* |
| CL8047.Contig1_All | 1.17 | 0.01 | -6.87 | *Morus mongolica* |
| Unigene20247_All | 0.62 | 0.01 | -5.95 | *Chenopodium quinoa* |
| Unigene22784_All | 0.42 | 0.01 | -5.39 | *Portulaca oleracea* |
| Unigene8514_All | 1.12 | 0.28 | -2.00 | *Chenopodium quinoa* |
| Unigene7008_All | 6.11 | 1.28 | -2.26 | *Vitis vinifera* |
| **Thylakoid membrane ATP synthase** | | | | |
| CL1029.Contig1_All | 0.01 | 0.79 | 6.30 | *Medicago truncatula* |
| CL1018.Contig1_All | 0.01 | 0.02 | 1.00 | *Arabidopsis thaliana* |
| CL9622.Contig1_All | 2.08 | 1.00 | -1.06 | *Ricinus communis* |
| **Carbon fixation** | | | | |
| Unigene26311_All | 0.01 | 0.53 | 5.73 | *Mesembryanthemum crystallinum* |
| CL1287.Contig1_All | 10.75 | 80.30 | 2.90 | *Spinacia oleracea* |
| Unigene10116_All | 0.55 | 2.19 | 1.99 | *Theobroma cacao* |
| CL6958.Contig4_All | 1.08 | 3.34 | 1.63 | *Ricinus communis* |
| CL791.Contig2_All | 0.68 | 2.04 | 1.58 | *Nelumbo nucifera* |
| Unigene12193_All | 0.21 | 0.63 | 1.58 | *Vernicia fordii* |
| Unigene6890_All | 2.06 | 4.83 | 1.23 | *Vitis vinifera* |
| CL4619.Contig1_All | 7.45 | 17.16 | 1.20 | *Eucalyptus grandis* |
| Unigene34895_All | 0.66 | 1.33 | 1.01 | *Rhododendron micranthum* |
| Unigene10436_All | 62.35 | 27.94 | -1.16 | *Beta vulgaris* |
| **Chlorophyll biosynthesis** | | | | |
| CL8468.Contig2_All | 0.03 | 7.18 | 7.90 | *Spinacia oleracea* |
| Unigene31043_All | 0.54 | 1.31 | 1.28 | *Spinacia oleracea* |
| **Chlorophyll catabolism** | | | | |
| Unigene14870_All | 0.22 | 1.68 | 2.93 | *Chenopodium album* |

**Table S11** Expression pattern validation of 30 randomly selected genes in leaves of *A. canescens* under 100 mM NaCl for 6 and 24 h by qRT-PCR.

| **Gene ID** | **Log_2_ Ratio** **(SL6/CL6)**  RNA-Seq qPCR | | **log_2_ Ratio** **(SL24/CL24)**  RNA-Seq qPCR | | **Homologous gene** |
| --- | --- | --- | --- | --- | --- |
| CL5945.Contig1_All | 1.19 | 1.15 | 0.05 | 0.86 | CCC1 |
| CL3698.Contig4_All | 6.04 | 5.05 | 3.17 | 4.65 | NRT2.5 |
| Unigene9556_All | 2.51 | 1.67 | 0.31 | 0.70 | P-H^+^ ATPase |
| CL8183.Contig1_All | 1.54 | 0.38 | 0.82 | 1.05 | V-CHX2 |
| CL4556.Contig4_All | 2.76 | 1.02 | -0.01 | 0.79 | VP1 |
| Unigene2433_All | -1.19 | -2.58 | 0.39 | 0.80 | NHX1 |
| CL5512.Contig2_All | 0.97 | 1.39 | 9.87 | 7.06 | SOS1 |
| CL539.Contig1_All | 7.27 | 5.56 | —— | 0.06 | HKT1 |
| Unigene11233_All | 6.70 | 5.36 | 4.17 | 5.41 | NIP5-1 |
| CL969.Contig1_All | 1.68 | 1.44 | 0.21 | 0.47 | PIP1 |
| Unigene14970_All | 1.64 | 0.90 | 0.15 | 0.43 | KEA5 |
| Unigene503_All | -2.71 | -3.52 | 0.40 | 0.55 | KUP12 |
| CL6985.Contig2_All | 10.05 | 7.09 | 1.61 | 1.35 | AKT1 |
| CL3066.Contig1_All | 5.24 | 3.93 | 0.22 | 1.35 | SKOR |
| CL5611.Contig3_All | 13.25 | 7.64 | 2.58 | 1.54 | PHT1-3 |
| CL1899.Contig5_All | 10.32 | 3.50 | 0.84 | 1.14 | CNGC |
| CL6653.Contig1_All | 9.72 | 8.38 | -3.17 | -1.38 | STAS1.3 |
| Unigene12189_All | 9.62 | 9.40 | —— | -1.64 | SLAH1 |
| Unigene1346_All | -9.49 | -4.47 | 0.26 | 1.13 | AMT1.2 |
| CL9305.Contig3_All | 2.34 | 3.39 | 0.51 | -0.26 | ProDH |
| CL8998.Contig2_All | -0.08 | 0.60 | 6.79 | 5.54 | BADH |
| CL9208.Contig1_All | 3.60 | 4.82 | 0.41 | 0.59 | NAC |
| Unigene9476_All | 6.23 | 7.29 | -5.00 | -5.13 | WRKY |
| CL3665.Contig2_All | 9.82 | 8.88 | -0.08 | -0.05 | POD |
| CL2142.Contig5_All | -3.04 | -4.13 | 0.58 | 0.31 | APX |
| CL5119.Contig1_All | -3.74 | -3.17 | 0.24 | -0.46 | SOD |
| CL1382.Contig4_All | 7.21 | 7.41 | 2.10 | 2.30 | GST |
| CL90.Contig1_All | -5.85 | -3.78 | 0.08 | -0.30 | Fd |
| CL2649.Contig3_All | 2.14 | 3.77 | 0.25 | 0.03 | PEPC |
| CL3000.Contig3_All | -7.13 | -8.23 | -0.27 | 0.21 | RubisCO |

**Table S12** Expression pattern validation of 30 randomly selected genes in roots of *A. canescens* under 100 mM NaCl for 6 and 24 h by qRT-PCR.

| **Gene ID** | **Log_2_ Ratio** **(SR6/CR6)**  RNA-Seq qPCR | | **log_2_ Ratio** **(SR24/CR24)**  RNA-Seq qPCR | | **Homologous gene** |
| --- | --- | --- | --- | --- | --- |
| CL5945.Contig1_All | -0.52 | 0.03 | -0.41 | -1.10 | CCC1 |
| CL3698.Contig4_All | -8.10 | -3.15 | -0.92 | -1.04 | NRT2.5 |
| Unigene9556_All | -2.01 | -0.92 | -0.28 | -0.91 | P-H^+^ ATPase |
| CL8183.Contig1_All | -2.01 | -1.77 | -0.29 | -0.59 | V-CHX2 |
| CL4556.Contig4_All | -2.85 | -1.36 | 0.25 | 0.92 | VP1 |
| Unigene2433_All | 1.09 | 2.33 | 0.34 | 1.02 | NHX1 |
| CL5512.Contig2_All | -2.78 | -1.19 | 1.20 | 1.52 | SOS1 |
| CL539.Contig1_All | -3.34 | -1.44 | -2.52 | -3.51 | HKT1 |
| Unigene11233_All | -5.64 | -4.80 | -0.57 | -0.81 | NIP5-1 |
| CL969.Contig1_All | -1.02 | -0.73 | -0.12 | -0.77 | PIP1 |
| Unigene14970_All | -1.55 | -1.36 | 0.08 | -0.51 | KEA5 |
| Unigene503_All | 2.86 | 3.39 | 0.23 | 1.17 | KUP12 |
| CL6985.Contig2_All | -9.88 | -6.44 | 5.37 | 4.91 | AKT1 |
| CL3066.Contig1_All | -6.68 | -4.94 | -0.01 | -1.21 | SKOR |
| CL5759.Contig2_All | -2.29 | -1.16 | -0.08 | -0.63 | CCX4 |
| CL5611.Contig3_All | -12.94 | -5.61 | -0.19 | -0.67 | PHT1-3 |
| CL1899.Contig5_All | -3.53 | -2.67 | 0.04 | -0.41 | CNGC |
| CL6653.Contig1_All | -11.85 | -7.70 | 0.00 | -0.55 | STAS1.3 |
| Unigene12189_All | -9.40 | -8.01 | 0.08 | 0.04 | SLAH1 |
| Unigene1346_All | 7.13 | 7.08 | 1.85 | 1.77 | AMT1.2 |
| CL9305.Contig3_All | -2.65 | -1.58 | -0.86 | -0.83 | ProDH |
| CL8998.Contig2_All | 3.16 | 1.54 | 0.99 | -0.15 | BADH |
| CL9208.Contig1_All | -3.17 | -1.76 | -1.40 | -2.32 | NAC |
| CL1223.Contig2_All | -3.50 | -1.28 | 0.16 | -0.36 | MYB |
| Unigene9476_All | -7.25 | -4.83 | -0.33 | -0.96 | WRKY |
| Unigene5013_All | -11.38 | -10.55 | -0.24 | -0.74 | GLP |
| CL3665.Contig2_All | -9.60 | -7.98 | 0.17 | -0.13 | POD |
| CL2142.Contig5_All | 1.89 | 3.64 | -0.09 | 0.06 | APX |
| CL5119.Contig1_All | 0.44 | 1.19 | -0.13 | -0.21 | SOD |
| CL1382.Contig4_All | -14.57 | -8.52 | -0.23 | -0.52 | GST |
